# Evaluation of 3D biomimetic microcarriers for enhancing therapeutic efficacy of human umbilical cord mesenchymal stem cells in psoriasis treatment

**DOI:** 10.3389/fimmu.2026.1687424

**Published:** 2026-02-03

**Authors:** XueMei Li, YanMei Chen, JiaWei Cai, YuQiong Huang, XiangLong Chen, MingYu Yu, Li Fu, Bao Chai, Cheng Zhang, ZhiYong Zhang, HongXiang Chen

**Affiliations:** 1 Department of Dermatology, Huazhong University of Science and Technology Union Shenzhen Hospital, Shenzhen, China; 2 Department of Dermatology, Shenzhen Second People’s Hospital/the First Affiliated Hospital of Shenzhen University Health Science Center, Shenzhen, China; 3 Translational Research Centre of Regenerative Medicine and 3D Printing, The Third Affiliated Hospital of Guangzhou Medical University, Guangzhou, Guangdong, China; 4 Department of Dermatology, Union Hospital, Tongji Medical College, Huazhong University of Science and Technology, Wuhan, China; 5 Guangdong Province Key Laboratory of Regional Immunity and Diseases, Department of Pharmacology and Shenzhen University International Cancer Center, Shenzhen University Medical School, Shenzhen, China; 6 Department of Dermatology, The 6th Affiliated Hospital of Shenzhen University Health Science Center, Shenzhen, China; 7 Department of Dermatology, Zhongnan Hospital of Wuhan University, Wuhan University, Wuhan, China

**Keywords:** hUC-MSCs, IL-17/NF-kB signaling, microcarrier-based 3D culture, psoriasis, stemness

## Abstract

Mesenchymal stem cells (MSCs) hold promise for regenerative medicine due to their unique biological properties, including self-renewal and multi-lineage differentiation potential. Conventional two-dimensional (2D) culture systems may hinder therapeutic efficacy due to challenges in maintaining quality and producing a sufficient quantity of cells for clinical applications. This study aimed to evaluate the influence of a three-dimensional (3D) microcarrier-bioreactor system on the biological characteristics of human umbilical cord MSCs (hUC-MSCs) and their potential therapeutic efficacy in a psoriasis mouse model. The 3D microcarrier-bioreactor system was observed to improve hUC-MSCs attachment and proliferation while preserving genetic stability, characteristic surface marker expression, non-tumorigenic properties, and differentiation potential, consistent with outcomes from 2D cultures. Moreover, the 3D-hUC-MSCs demonstrated enhanced proliferation, stemness, immune function, and cell viability compared to those cultured in 2D systems.*In vitro* experiments demonstrated that 3D-hUC-MSCs supernatants effectively suppressed IL-17A-induced NF-κB signaling in keratinocytes. *In vivo*, 3D-hUC-MSCs significantly reduced IMQ+IL-23-induced psoriasis-like skin inflammation by reducing immune cell infiltration and inhibiting IL-17-associated inflammatory pathways. Transcriptomic analysis revealed that 3D-hUC-MSCs modulated signaling pathways associated with inflammation and innate immune responses. Our findings suggest that the 3D microcarrier-bioreactor system holds promise as a strategy to enhance the therapeutic potential of hUC-MSCs, particularly in the treatment of immune-mediated disorders such as psoriasis.

## 1 Introduction

Psoriasis is a chronic inflammatory skin disorder that affects approximately 2%–3% of the global population and can occur at any age; it has a significant impact on quality of life, especially when critical areas like the face, palms, soles, and genitalia are involved (1). Characterized by abnormal keratinocyte proliferation and immune cell infiltration, psoriasis involves disrupted innate and adaptive immune responses involving T cells, keratinocytes, neutrophils, and dendritic cells (2). Genetic predispositions, infections, stress, or trauma can trigger immune responses, leading to cytokine production [e.g., Interleukin 23 (IL-23), Tumor Necrosis Factor Alpha (TNF-α)] that exacerbates the disease. Current biologic therapies, despite their efficacy, may cause relapse, resistance, and adverse effects, highlighting the need for safer treatments (3).

Stem cell therapy, particularly using mesenchymal stem cells (MSCs), shows promise for treating various chronic diseases, including cancer, organ failure, and autoimmune conditions such as psoriasis (4, 5). Traditional treatments for these diseases often struggle with immunological rejection, donor shortages, and high medical costs; by contrast, MSCs are multipotent, are capable of differentiating into multiple cell types, and possess significant immunomodulatory abilities without causing severe immune reactions, making them a promising treatment option for severe illnesses.

Recent studies suggest that umbilical cord MSCs (UMSCs) are safe and effective in treating psoriasis, improving lesion severity, and modulating immune cell populations. UMSCs transplantation increases the frequency of regulatory T cells (Tregs) and CD4+ memory T cells, while decreasing Th17 and CD4+ naïve T cells in the peripheral blood of some psoriasis patients (6). MSCs modulate immune cells through cell-to-cell contact or paracrine mechanisms, involving factors such as iNOS, IDO, FasL, PD-L1, galectin-1, HO-1, ICOSL, and HLA-G5 (7). These factors suppress T and B cell activation while promoting Treg differentiation, making UMSCs strong candidates for psoriasis treatment (8).

Cell culture is a crucial and necessary process in stem cell research. Traditionally, a 2D approach has been widely employed. However, 2D cell culture has limitations in simulating natural and extracellular matrix (ECM) components, failing to accurately replicate pH, oxygen, and nutrient levels of the *in vivo* environment. In addition, cells in 2D culture undergo morphological changes and cannot effectively mimic *in vivo* cell-to-cell and cell-to-ECM interactions (9). To address these limitations, various 3D culture techniques have been developed, including spheroid culture, hanging drop (HD) culture, ultra-low attachment (ULA) plate culture, suspension culture, and ECM-based models (10–16). Compared with 2D culture, 3D culture has many advantages and is widely used in stem cell research, cancer research, and drug discovery (17–19). Cells cultured in 3D show significant improvements in morphology, cell number, protein synthesis, differentiation, proliferation, and response to stimuli. In addition, 3D culture leads to higher pluripotency and differentiation potential in MSCs. However, MSCs may lose their ability to inhibit immune responses after forming spheroids.

Despite the promising therapeutic potential of hUC-MSCs, the effects of a microcarrier-based 3D culture system on their biological properties and immunomodulatory functions remain insufficiently characterized. In this study, we systematically compared conventional 2D culture with a novel 3D microcarrier approach for hUC-MSCs, evaluating cell morphology, viability, senescence, differentiation potential, and expression of key immunomodulatory factors. Furthermore, we investigated the *in vivo* therapeutic efficacy of these cells in an imiquimod- and IL-23-induced psoriasis mouse model. Our findings provide mechanistic insights into how 3D culture may enhance the immunomodulatory and therapeutic properties of hUC-MSCs, offering potential strategies to optimize MSC-based therapies for psoriasis.

## 2 Materials and methods

### 2.1 2D monolayer culture of hUC-MSCs

Human umbilical cords were obtained from healthy full-term infants delivered by natural birth or cesarean section at The Third Affiliated Hospital of Guangzhou Medical University. The volunteers provided written informed consent. The cords were delivered to the laboratory in a sterilized jar filled with cold phosphate-buffered saline (PBS). The isolation and primary culture of hUC-MSCs were performed according to the tissue block adhesion method. The mesenchymal tissue was minced into small pieces (1–8 cm^3^), evenly spread in a T175 flask, and cultured in serum-free hUC-MSCs culture UltraMedia (RGM0051, REGEN-αGEEK Biotechnology Co., Ltd., Haining, China). The medium was replaced with fresh complete medium every 2–3 days, and the hUC-MSCs were split at 90% confluence. The primary cells were harvested for subculture. Around 1.4 ×10^6^ P3 hUC-MSCs were inoculated into T175 for monolayer culture with serum-free hMSC culture medium; they were grown to 90% confluence and harvested with UltraTryple (RGM0061, REGEN-αGEEK Biotechnology Co., Ltd., Haining, China).

### 2.2 Cell inoculation and expansion on 3D biomimetic microcarrier^®^

The 3D Biomimetic microcarriers were obtained from Haining REGEN-αGEEK Biotechnology Co., Ltd. The microcarriers are primarily composed of recombinant humanized collagen and range in size from 125 to 250 μm, featuring a porosity of ≥90% and pore diameters of ≥20 μm [China’s Centre for Drug Evaluation (CDE) registration number: F20230000567]. A total of 100–125 mg 3D Biomimetic microcarriers (RC001LE) were added to a sterile 125 mL spinner flask (mBioR-Ves-125-CC, REGEN-αGEEK Biotechnology Co., Ltd., Haining, China) with 30 mL serum-free hUC-MSCs culture UltraMedia (RGM0051, REGEN-αGEEK Biotechnology Co., Ltd., Haining, China) to agitate and disperse fully. A total of 2 × 10^6^ P2 hUC-MSCs were inoculated into the flask, and the medium was immediately added to a final volume of 50 mL and fully mixed with the microcarriers. The flasks were then placed on a 3D mini BioR system inside a 37°C, 5% CO_2_ incubator and with different agitation protocols programmed. The cells were inoculated on day 0 according to 24 cycles and cultured in intermittent cycles of 35 rpm × 5 min and 0 rpm × 1–2 h. After a 24-hour inoculation period, agitation was set to a constant 40 rpm, and medium was added to a final volume of 75 mL. Aliquots of 1 mL were taken from the side arm with a pipette at 1, 3, and 4 days after inoculation to monitor cell growth. All cells were cultured for 5 days following this protocol. Then, 3g microcarriers laden with P3 6 × 10^7^ cells were then transferred to a 2 L bioreactor to obtain a higher cell quantities. According to the manufacturer’s instructions, the 3D culture parameters were programmed as follows: temperature: 37°C, pH: 7.3, oxygen level: 40%. The initial agitation (on inoculation day) was set at 50 rpm for 5 min, followed by a 30-min period with no agitation. From day 1 to day 7, the agitation speed was maintained at 60 rpm. The serum-free medium was supplemented to 2 L after 24 h, and subsequently, fresh medium was replaced 50% of the medium every 48 hours before harvesting cells on day 7.

### 2.3 Cell harvesting and counting

3D-hUC-MSC-laden microcarriers were precipitated to the bottom of the centrifuge tube, and the supernatant was carefully removed to ensure no loss of microcarriers. A 3D Microcarrier Lysis Compound (MC001LS, REGEN-αGEEK Biotechnology Co., Ltd., Haining, China) was added at a ratio of 0.2 mL/mg microcarrier and incubated at 37°C for 30 min with an agitation speed of 50 rpm, which was gently mixed with a pipette every 10 min to accelerate microcarrier and cell lysis. Cell number and viability were recorded by Countstar AO/PI dye using a cell counter (Rigel S2, Countstar, Shang Hai, China). hUC-MSCs were used for assays within no more than six passages.

### 2.4 Fluorescence staining of cells on microcarriers

3D-hUC-MSC-laden microcarriers were precipitated to the bottom of the wells of 48-well plates, and the supernatant was carefully removed to ensure no loss of microcarriers. Microcarriers were stained with a Calcein-AM/PI Cell Viability Assay Kit (C2015M, Beyotime, China) following the manufacturer’s instructions, incubated at 37°C for 30 min without light. Fluorescent images were acquired using a fluorescence microscope (OLYMPUS IX83, Japan).

### 2.5 Analysis of surface antigens of hUC-MSCs

3D-hUC-MSCs were harvested and then incubated directly with indicators for positive markers (CD73-PE, CD90-PE, and CD105-APC; Biolegend, USA) as well as negative markers (CD11b-FITC, CD19-FITC, CD45-FITC, CD34-PE, and HLA-DR-PerCP; Elabsciece, USA). A total of 5 × 10^4^ events were acquired by flow cytometry (Attune^®^ NxT, Thermo Fisher) and analyzed using FlowJo The V7.6.2 software (BD Biosciences).

### 2.6 Differentiation of hUC-MSCs

Adipogenic (HUXUC-90031), osteogenic (HUXUC-90021), and chondrogenic (HUXUC-90041) differentiation and characterization kits were purchased from OriCell. Dissociated 2D- or 3D-hUC-MSCs were collected, reseeded into 6-well plates, and cultured using respective culture and characterization kits to induce adipogenic, osteogenic, and chondrogenic differentiation. All protocols were performed following the manufacturer’s instructions.

### 2.7 Karyotype analysis

hUC-MSCs harvested from 2D monolayer culture (in the exponential phase in T175) or 3D Biomimetic microcarriers^®^ (in the exponential phase in 125 mL spinner flasks) were cryopreserved and sent to a certified third-party laboratory (KingMed Diagnostics, Guangzhou, China) for standard chromosome analysis at 300-band resolution following standard procedures.

### 2.8 Short tandem repeat analysis

3D-hUC-MSCs were harvested, cryopreserved, and then sent to a certified third-party laboratory (Procell Life Science, Wuhan, China) for PCR testing. STR data analysis was performed using the ExPASy database.

### 2.9 Telomerase activity assay

RNA (1 μg) was extracted from 3D-hUC-MSCs using the Ultrapure RNA Kit (CWBIO, Jiangsu, China). Telomerase activity assay was performed using a telomerase activity TERT real-time quantitative detection kit (BPT50, Biowing, China) following the manufacturer’s instructions.

### 2.10 Morphology of UC-MSCs

3D-hUC-MSCs were further cultured in T75 flasks. The morphological images were obtained by microscopy on day 3 of culture.

### 2.11 RT-qPCR

Total RNA from 2D-hUC-MSCs, 3D-hUC-MSCs, Hacat cells (CTCC-0383-Luc1, ZhejiangMeisen Cell Technology, China), and skin tissues was extracted according to the protocol of the Ultrapure RNA Kit (CWBIO, Jiangsu, China), and RNA concentration was determined using NanoDrop (Thermo Fisher, USA). RNA extracts (1 μg) were reverse-transcribed into complementary DNA (cDNA) according to the protocol of the Evo M-MLV Reverse Transcription Premixed Kit Ver.2 (Accurate Biotechnology (Hunan) Co., Ltd., Changsha, China). QRT-PCR was run following the protocol of the SYBR Green qPCR Kit (AG11718, Accurate Biotechnology (Hunan) Co., Ltd., Changsha, China) and using the QuantStudio 3 Real-Time PCR System (Thermo Fisher, USA) with each reaction run in triplicate. The comparison threshold cycle (Ct) method was used to measure the analysis results and fold changes. The RT-qPCR primers for target genes were designed using Primer Bank (**Supplementary Table S1**).

### 2.12 Soft agar colony formation assay

Agarose solutions of 0.7% and 1% were prepared with ultrapure water and agarose (Biofroxx, Germany) and autoclaved at 121°C for 20 min. The 1% agarose solution was mixed with an equal volume of 2× DMEM (VivaCell Biosciences, Shanghai, China) containing 20% FBS (209111, NEST Biotechnology, Wuxi, China), quickly added to a 6-well plate (4 mL/well) to create the bottom layer of agar, and solidified at room temperature. The 0.7% agarose solution was mixed with an equal amount of 2× DMEM containing 20% FBS to create a 0.35% agarose mixture. A total of 5000 cells/well (harvested from 2D and 3D cultures) were mixed with the 0.35% agarose mixture and evenly plated on top of the agar base layer at a volume of 2 mL per well. HeLa cells were used as a positive control. After solidification at room temperature, the dishes were placed in a 37 °C, 5% CO2 incubator for 16 days and observed under a microscope. Assays were set up in triplicate.

### 2.13 Colony-forming unit-fibroblast assay

2D- and 3D-hUC-MSCs were seeded in 10 cm culture dishes at a density of 1000 cells/per dish and placed in a 37 °C, 5% CO2 incubator. After culture for 14 days, the colonies were fixed with 4% paraformaldehyde (Solarbio, Beijing, China) for 10 min at room temperature. Colonies were stained with 2.5% Crystal Violet for 10 min at room temperature. The dishes were washed with PBS three times and dried, then photographed with the same exposure settings. Colonies larger than 2 mm in diameter were counted and analyzed using ImageJ.

### 2.14 Senescence assay

2D- and 3D-hUC-MSCs were seeded in six-well plates at a density of 5 × 10^4^ cells/well and cultured to 80%–90% confluence. Cell senescence was detected using the Cell Senescence β-Galactosidase Staining Kit (C0602, Beyotime, Shanghai, China). The results were observed with a microscope and recorded, and the stained fractions were quantitatively counted using Image J. SA-β-gal positive cells appeared blue.

### 2.15 Protein isolation and western blot analysis

Cells were collected via centrifugation and lysed using RIPA buffer (Solarbio). After thorough pipetting, the lysate was centrifuged at 15,000 rpm for 15 minutes. Total protein concentration was determined with the BCA protein assay kit (Thermo Fisher Scientific). The samples were then analyzed by sodium dodecyl sulfate-polyacrylamide gel electrophoresis (SDS-PAGE), transferred onto a nitrocellulose membrane, and incubated with specific antibodies overnight at 4°C with gentle shaking. Subsequently, the blots were treated with a peroxidase-conjugated secondary antibody for 1 hour at room temperature, followed by detection using enhanced chemiluminescence (Intron Biotechnology). The primary antibodies used included β-actin (Santa Cruz Biotechnologies, Santa Cruz, CA, USA), p-P65/P65 (Cell Signaling Technology, Danvers, MA, USA), IL-17RA (Thermo Fisher Scientific), and IL-17A (Abcam, Cambridge, UK).

### 2.16 Mice

Male C57BL/6-Mice (18–20 g) at 6–8 weeks of age were purchased from the Medical Laboratory Animal Center(Guangdong, China). The animals were fed with a standard laboratory diet and water under specific conditions (12-h light-dark rhythm, 23 ± 2°C ambient temperature). They were acclimatized for at least 7 days before use. All experimental protocols were conducted in accordance with the ARRIVE guidelines and were approved by the animal care and use committee of Shenzhen TopBiotech limited company (Shenzhen, China, NO.TOP-IACUC-2022-0209). Mice were anesthetized with isoflurane (2–4% for induction and 1–2% for maintenance, in oxygen) using a precision vaporizer prior to experimental procedures. At the end of the experiments, animals were euthanized by cervical dislocation under deep anesthesia.

### 2.17 Experimental psoriasis mouse models

Male C57BL/6 mice (7weeks old,18-20g) were randomly divided into 4 groups (*n* = 5). Mice were topically treated with a dose of 42 mg 5% IMQ cream (Mingxin Pharmaceuticals, Sichuan, China) in the morning and injected subcutaneously with IL-23(0.5 μg in 50 μL PBS) in the afternoon on their shaved dorsal skin for 7 consecutive days. Prior to each IL-23 injection, mice were briefly anesthetized with isoflurane (induction 3–4%, maintenance 1.5–2%) delivered via a calibrated vaporizer to minimize discomfort.Control groups received petroleum jelly topically and 50μL PBS subcutaneously. MSC treatment groups received 2D-hUC-MSC or 3D-hUC-MSCs (5 × 10^5^ cells in 100 μL PBS) via tail vein injection on days 1 and 3; tail vein injection was selected to enable systemic delivery and assess overall immunomodulatory effects. The control and IMQ+IL-23 group received equivalent volumes of saline via tail vein injection at the same time points. The entire experiment was performed in duplicate. PASI scores of the severity of skin inflammation were assessed by two laboratory assistants blinded to the group information based on previous descriptions (43). On day 8, serum samples and skin tissues were collected from the sacrificed mice for subsequent studies. All mice were monitored daily for body weight, grooming behavior, activity, and skin condition, and animals exhibiting excessive distress were removed following humane endpoints approved by the Institutional Animal Care and Use Committee (IACUC No. TOP-IACUC-2022-0209).

### 2.18 Histology and immunohistochemistry staining

Universal tissue fixative was used for fixation; paraffin-embedded tissue sections were 4 µ thick and mounted on glass slides for various stains. Some sections were stained with hematoxylin-eosin (H&E) to evaluate epidermal thickness. For immunochemistry, some skin paraffin sections were stained with anti-Ki67 (1:50), anti-CD3 (ab237721; Abcam, Cambridge, UK), anti-Ly6G (ab238132; Abcam, Cambridge, UK), or anti-F4/80 (70076; Cell Signaling Technology, Danvers, MA, USA). Pathological images were taken with an upright microscope (Olympus, Japan) and analyzed using ImageJ.

### 2.19 Flow cytometry analysis

Single cell suspensions were obtained by isolating lymph nodes from individual mice and passing the single cell suspensions of lymph nodes through 40 μm sterile mesh. For cell surface antigen staining, cells were pre-blocked with Fc receptors (BD, Pharmingen) for 10 min at 4°C. Wash twice with PBS, and incubate the surface with APC-CD3 (557832; BD, Pharmingen), BV421-CD4 (562424; BD, Pharmingen) γδT (562892; BD, Pharmingen) antibodies in a dark room at 4°C for 1 hour. For intracellular staining, cells were fixed in 4X Fix/Perm concentrate for 30 minutes at room temperature. After washing with Perm/Wash buffer, cells were incubated with PE-T-bet (2410093; Invitrogen) and PE-RORγT (2306905; Invitrogen) antibodies overnight at 44°C. Then wash twice with cold PBS, resuspend the cells in stain Buffer, and analyze using BD FACS canto II flow cytometer and FlowJo.

### 2.20 Cytokine detection by enzyme-linked immunosorbent assay

Cytokines from mouse serums were assayed by ELISA kits following the manufacturers’ instructions (RK04288; Abclonal,USA).

### 2.21 RNA-sequencing

Total RNA was extracted from both IMQ-induced psoriasis mice and treatment group tissues using the Trizol method, following the manufacturer’s protocol. For library construction, mRNAs with polyA tails were enriched using Oligo(dT) magnetic beads and fragmented into small pieces. First-strand cDNA was synthesized using random hexamer primers, followed by second-strand cDNA synthesis incorporating dUTPs for strand specificity. End repair and dA-tailing were performed concurrently. Sequencing adapters were ligated to the cDNA fragments, and post-ligation, DNA magnetic beads were used for purification and fragment selection to yield a library with insert sizes of 250–350 bp. The ligated products were then amplified by PCR, purified, and solubilized in nuclease-free water. Library quality was assessed using a Qubit fluorometer and a Qsep400 high-throughput biofragment analyzer. The double-stranded DNA library was cyclized to obtain a single-stranded circular DNA library, which was amplified with phi29 to create DNA nanoballs (DNB) containing over 300 copies of DNA molecules. DNBs were loaded into the sequencing chip and sequenced on the BGI sequencing platform (DNBSEQ-T7). Raw sequencing reads were filtered and quality-trimmed using fastp, and aligned to the mouse reference genome. Differential expression analysis was performed using DESeq2, with significance thresholds set at adjusted p-value (FDR) < 0.05 and |log2 fold change| ≥1. Batch effects were evaluated and corrected using the sva package when necessary. Functional enrichment analyses, including Gene Ontology (GO) biological process and molecular function terms and KEGG pathway analysis, were conducted using clusterProfiler with FDR < 0.1. Volcano plots and heatmaps were generated to visualize differentially expressed genes.

### 2.22 Statistical analysis

All data are presented as mean ± SEM. Prior to statistical testing, the data distribution was evaluated using the Shapiro-Wilk test. For normally distributed data with homogeneous variance, comparisons among multiple groups were conducted using one-way ANOVA, followed by Tukey’s *post hoc* test. In contrast, for non-normally distributed data, the Kruskal-Wallis test, followed by Dunn’s multiple comparison test, was employed. Two-group comparisons were analyzed using either an unpaired two-tailed Student’s t-test (parametric) or the Mann-Whitney U test (non-parametric), contingent upon the data distribution. Multiple comparisons were controlled using Tukey’s or Dunn’s *post hoc* procedures to mitigate the inflation of type I error. The number of biological replicates (n) for each experiment is specified in the corresponding figure legends. The sample size for *in vitro* and *in vivo* assays was informed by previously published studies utilizing similar models and effect sizes; although formal power calculations were not performed, the replicate numbers employed in this study are standard in MSC and psoriasis research and are adequate for detecting biologically meaningful differences. All analyses were performed using GraphPad Prism 9.5, with a P value < 0.05 deemed statistically significant.

## 3 Results

**Figure 1:**
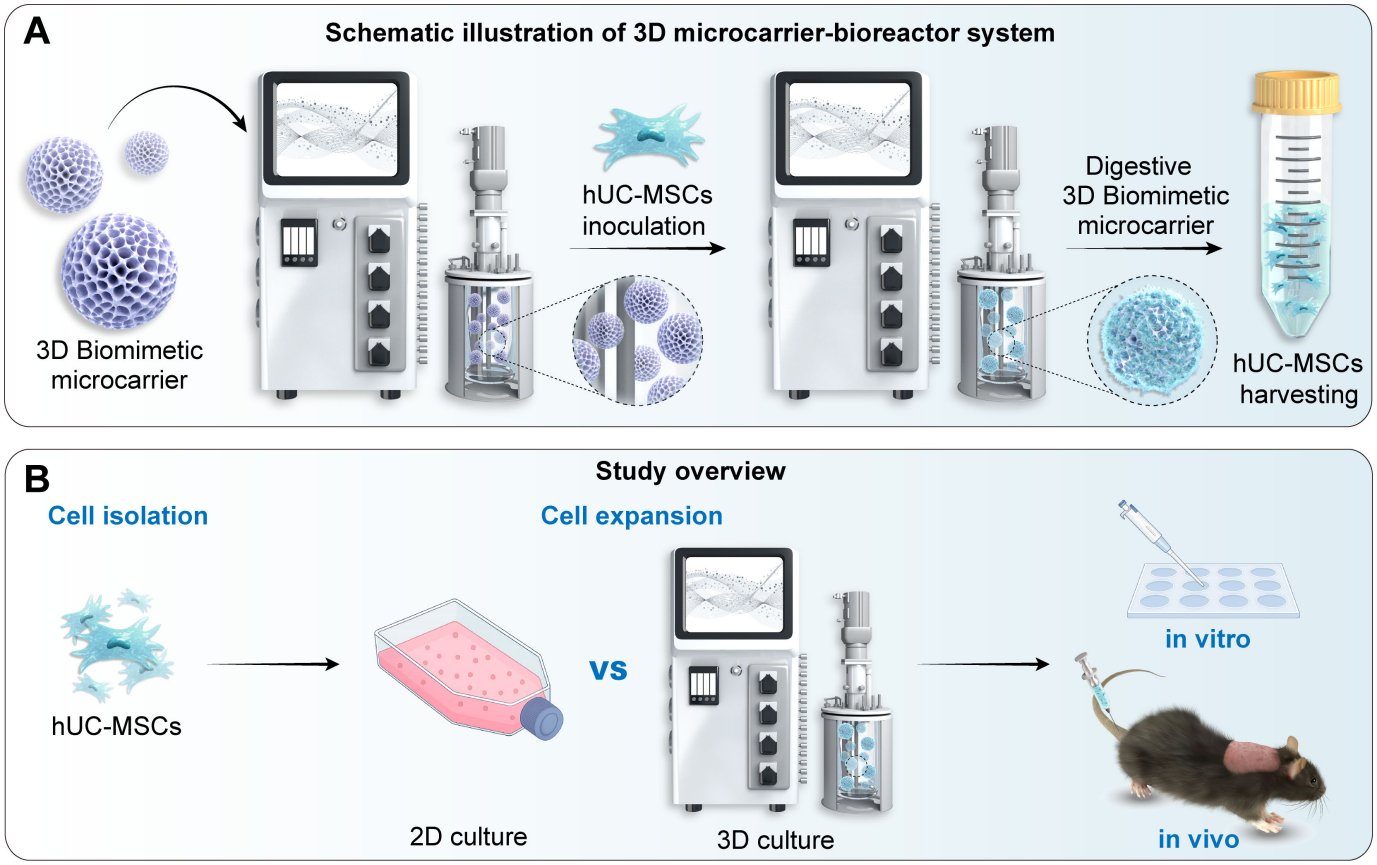
Schematic overview of the 3D microcarrier bioreactor system and study design. **(A)** Schematic illustration of the 3D biomimetic microcarrier–bioreactor culture system. hUC-MSCs were inoculated onto biomimetic microcarriers and expanded in a dynamic bioreactor environment, followed by enzymatic digestion of microcarriers and harvesting of 3D-expanded hUC-MSCs. **(B)** Overview of the experimental workflow. hUC-MSCs were isolated and expanded using either traditional 2D culture or the 3D microcarrier-bioreactor system. Expanded MSCs were subsequently used for *in vitro* assays and *in vivo* therapy in an IMQ + IL-23–induced psoriasis-like mouse model.

**Figure 1** provides a schematic overview of the experimental. We first expanded hUC-MSCs using a microcarrier-based 3D dynamic system, and then compared these 3D-expanded cells with hUC-MSCs cultured under conventional 2D conditions, examining their morphology, proliferation, senescence, differentiation capacity, and immunomodulatory characteristics. Subsequently, we evaluated the therapeutic efficacy of these cells both *in vitro* and in an imiquimod + IL-23–induced psoriasis mouse model.

### 3.1 Morphological changes and cell surface marker expression in 3D-hUC-MSCs

**Figure 2:**
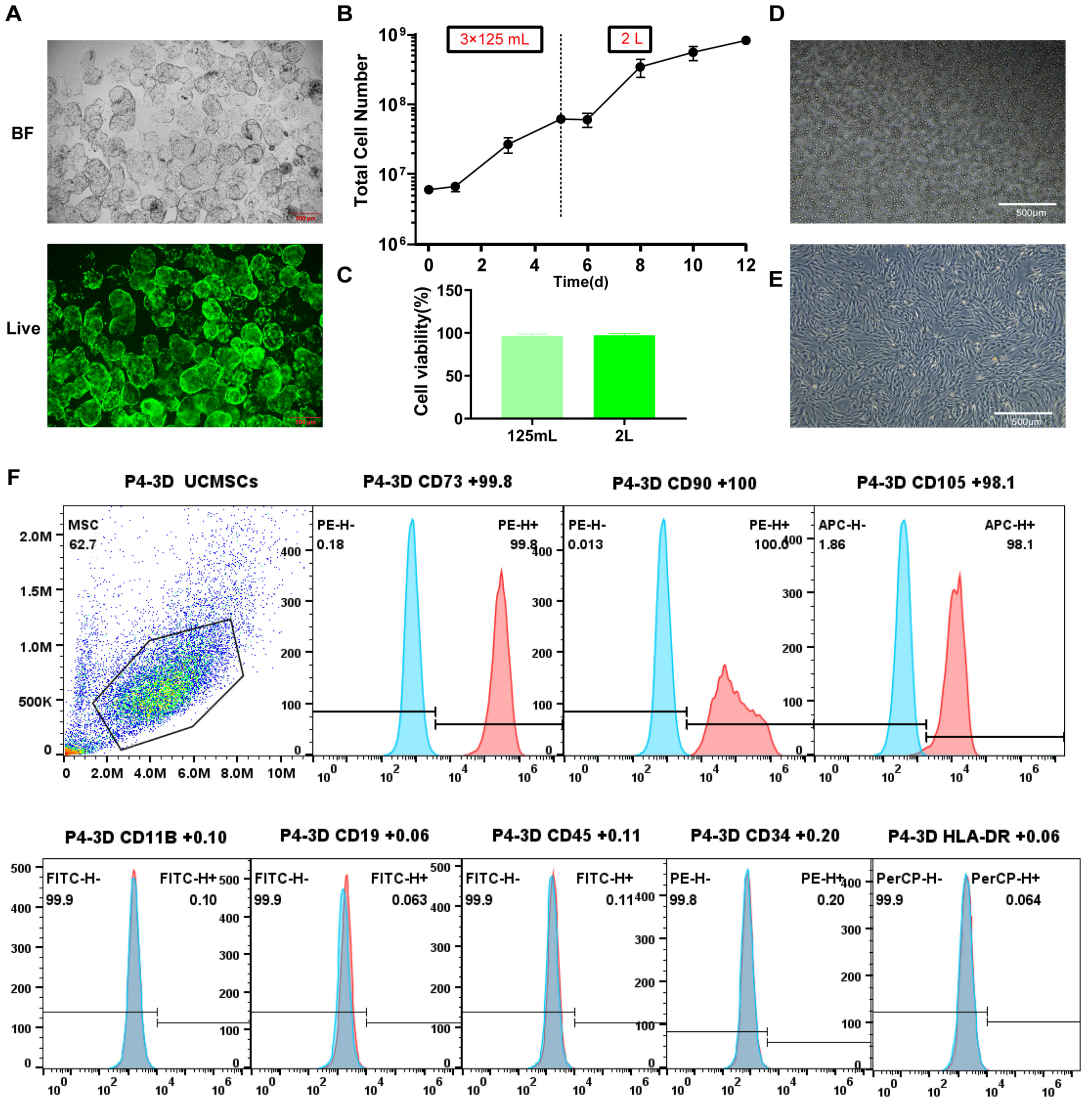
Morphological changes and cell surface marker expression in 3D-cultured MSCs. **(A)** Fluorescent images of live staining of MSCs on 3D-microcarriers. scale bar: 500 µm. **(B)** Growth curve. **(C)** Cell viability of UCMSCs harvested by the 125 mL, 2 L bioreactor. **(D)** Morphology of microcarrier residue; scale bar: 500 µm. **(E)** Morphology of 3D expanded UCMSCs; scale bar: 500 µm. **(F)** Flow cytometric analysis of cell surface marker expression in 3D-hUC-MSCs. Positive markers: CD73, CD90, and CD105; negative markers: CD11B, CD19, CD45, CD34, and HLA-DR. *n* = 3 independent assays, with each experiment comprising 3 technical replicates for every condition.

The hUC-MSCs were cultured in a monolayer until the third passage to prepare three-dimensional spheres. Fluorescence microscopy revealed that hUC-MSCs were attached to the 3D biomimetic microcarriers (**Figure 2A**). Using 3D Biomimetic microcarrier^®^ platform, we inoculated 2×10^6^ of UCMSCs into a 125 mL spinner flask and harvested approximately 2×10^7^,and then 6×10^7^ cells from three 125 mL bioreactors were transferred to a 2 L bioreactor, yielding a total of approximately 8.25×10^8^ cells (**Figure 2B**). The viability of harvested cells in the 125 mL, 2 L bioreactor (**Figure 2C**) were higher than 95%, without any significant difference between these two groups. After complete lysis of the 3D bionic microcarriers, the result showed that the residual concentration of microcarrier degradation was less than 1.5 μg/mL in each 1×10^7^ cells suspension, within the specified range, and no visible microcarrier fragments were observed under the microscope (**Figure 2D**). Besides, 3D expanded hUC-MSCs had good adhesion ability and exhibited a spindle morphology characteristic of fibroblasts, similar to cells cultured in 2D (**Figure 2E**). In addition, 3D-hUC-MSCs were positive for CD73, CD90, and CD105 expression and were negative for CD11b, CD19, CD34, CD45, and HLA-DR markers (**Figure 2F**). These findings suggest that 3D biomimetic microcarriers can enhance the attachment and proliferation of hUC-MSCs while maintaining desirable morphology and phenotypic expression.

### 3.2 Cell quality assessment of 3D-hUC-MSCs

**Figure 3:**
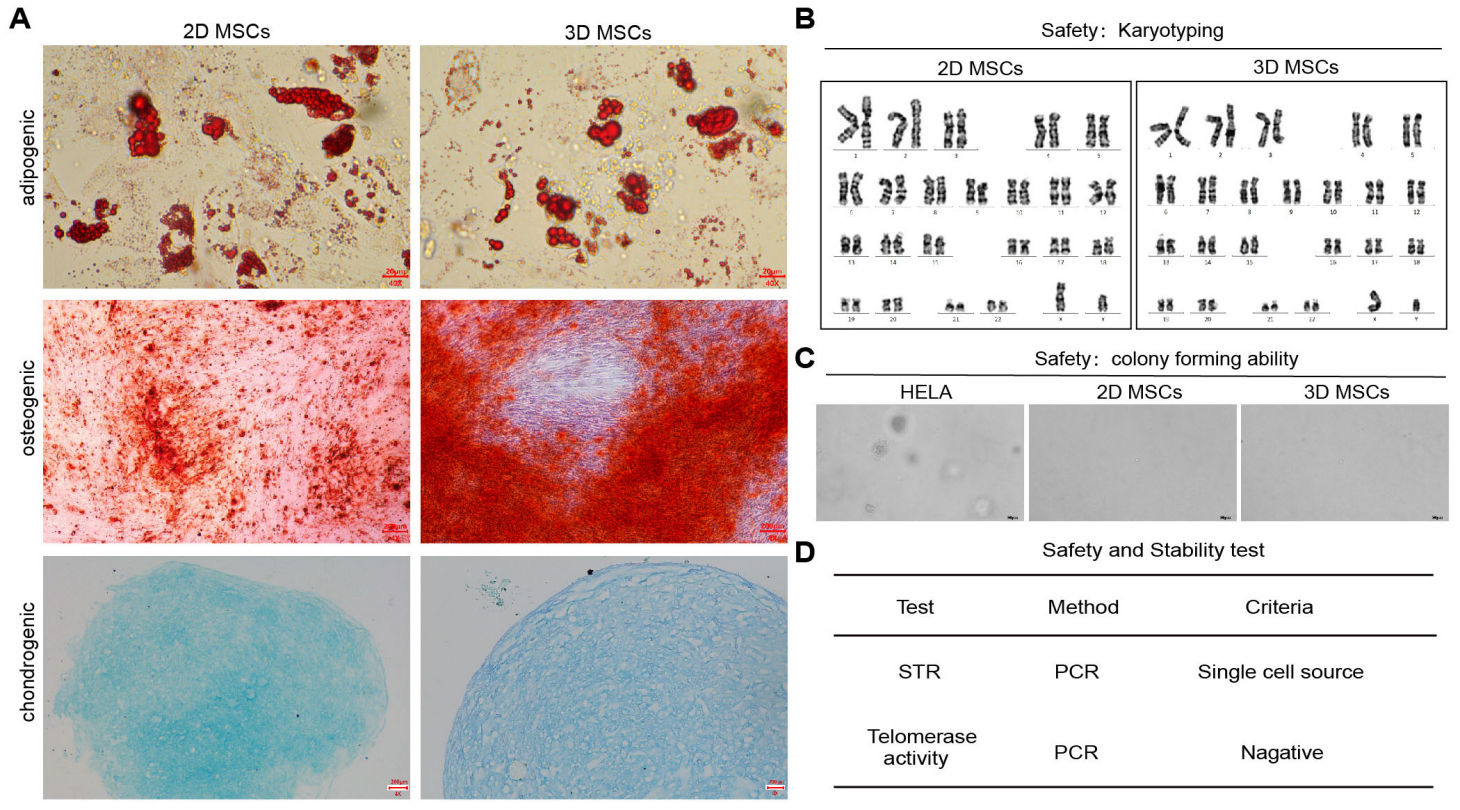
Quality assessment of 2D- and 3D-hUC-MSCs. **(A)** Multipotency of 3D expanded hUC-MSCs was tested by differentiation along adipogenic, osteogenic, and chondrogenic pathways; scale bars: 20 and 200 µm. **(B)** Karyotyping. **(C)** colony forming ability. **(D)** STR and telomerase activity of 3D-hUC-MSCs. *n* = 3 independent assays, with each experiment comprising 3 technical replicates for every condition.

Next, we evaluated the cell quality and safety of 3D-hUC-MSCs. 3D-hUC-MSCs maintained their ability to differentiate into adipogenic, osteogenic, and chondrogenic lineages (**Figure 3A**). Tumorigenesis is a prominent risk in the field of cell therapy, necessitating strong evidence of genetic stability and lack of tumorigenicity in cultured cells. Despite their high proliferation rate, karyotype analysis (**Figure 3B**) confirmed the genomic stability of 3D microcarrier-cultured cells; in addition, both the soft agar colony formation assay (**Figure 3C**) and telomerase activity assay (**Figure 3D**) demonstrated their lack of tumorigenicity. Short tandem repeat (STR) analysis (**Figure 3D**) also confirmed that hUC-MSCs were not contaminated by other cells. These results demonstrated that 3D culture conditions maintained the differentiation potential of hUC-MSCs into adipogenic, osteogenic, and chondrogenic lineages and that the quality of hUC-MSCs remained unaltered following both 2D and 3D culture.

### 3.3 Potency assays of 3D-hUC-MSCs

**Figure 4:**
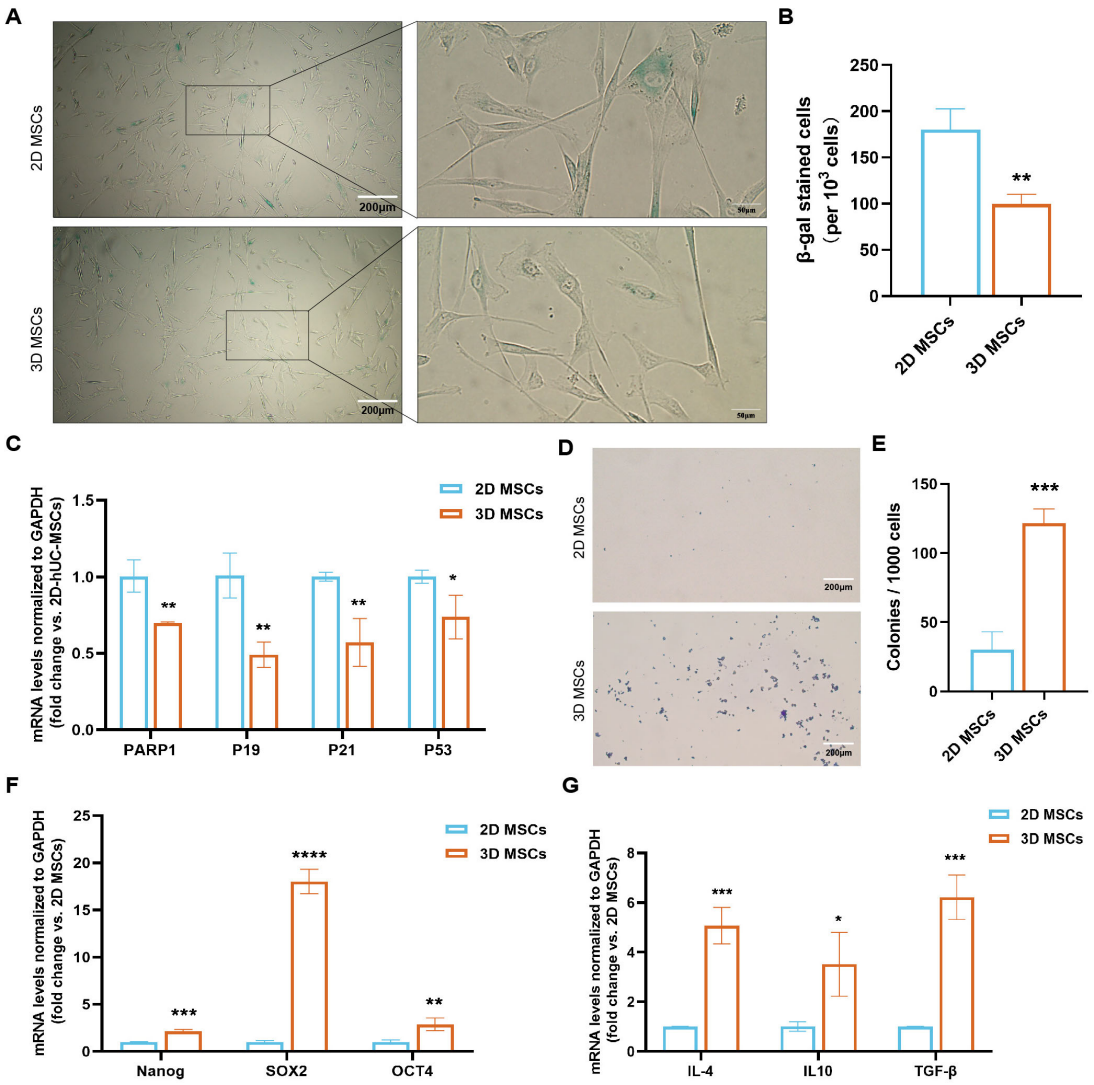
Cell senescence expression, viability, pluripotency, and immunomodulatory factor expression in 2D- and 3D-hUC-MSCs. **(A, B)** Cell senescence analyzed and quantified by staining 2D and 3D-cultured cells with β-galactosidase; scale bars: 200 and 50 µm. **(C)** Relative mRNA levels of senescence-related markers analyzed by RT-qPCR. **(D, E)** Crystal violet staining of CFU-F from 2D- and 3D-hUC-MSCs; scale bar: 200 µm. **(F)** Relative gene expression level of pluripotency markers analyzed by RT-qPCR. **(G)** Relative mRNA level of immunomodulatory or anti-inflammatory related markers analyzed by RT-qPCR. Data are represented as the mean ± SEM. Statistical significance was calculated by T-tests. *P < 0.05, **P < 0.01, ***P < 0.001, ****P < 0.0001. ns, not significant. *n* = 3 independent assays, with each experiment comprising 3 technical replicates for every condition.

To assess the impact of 3D culturing on cell activity, β-galactosidase activity and the mRNA expression of senescence-associated secretory phenotype markers were examined. The 3D-hUC-MSCs did not exhibit an increase in β-galactosidase activity, as indicated by SA-β-gal staining, when compared to 2D cultures (**Figures 4A, B**). Additionally, mRNA levels of PARP1, P19, P21, and P53 were lower in 3D-hUC-MSCs than in 2D-hUC-MSCs (**Figure 4C**). The number of fibroblast colony-forming units was significantly increased in 3D-cultured cells compared with 2D-cultured cells (**Figures 4D, E**).The impact of 3D culture conditions on the stemness of hUC-MSCs was evaluated using the expression of pluripotency markers. The expression levels of OCT4, SOX2, and NANOG were quantified using reverse-transcription quantitative PCR (RT-qPCR); hUC-MSCs cultured in 3D conditions exhibited higher mRNA expression of these pluripotency markers compared with those cultured in 2D conditions (**Figure 4F**). MSCs are considered promising candidates for the treatment of immune-mediated diseases due to their distinct immunomodulatory and anti-inflammatory properties. We quantitatively analyzed the expression of immunomodulatory factors IL-4, IL-10, and TGF-β secreted by hUC-MSCs in 2D and 3D cultures using RT-qPCR (**Figure 4G**). The increased expression of these immunomodulatory and anti-inflammatory factors in hUC-MSCs cultured in 3D conditions indicated that these cells were more effective in reducing inflammatory responses and modulating immune responses than those cultured in 2D conditions.

### 3.4 3D-hUC-MSC supernatants inhibited IL-17A-activated NF-κB signaling pathway *in vitro*

**Figure 5:**
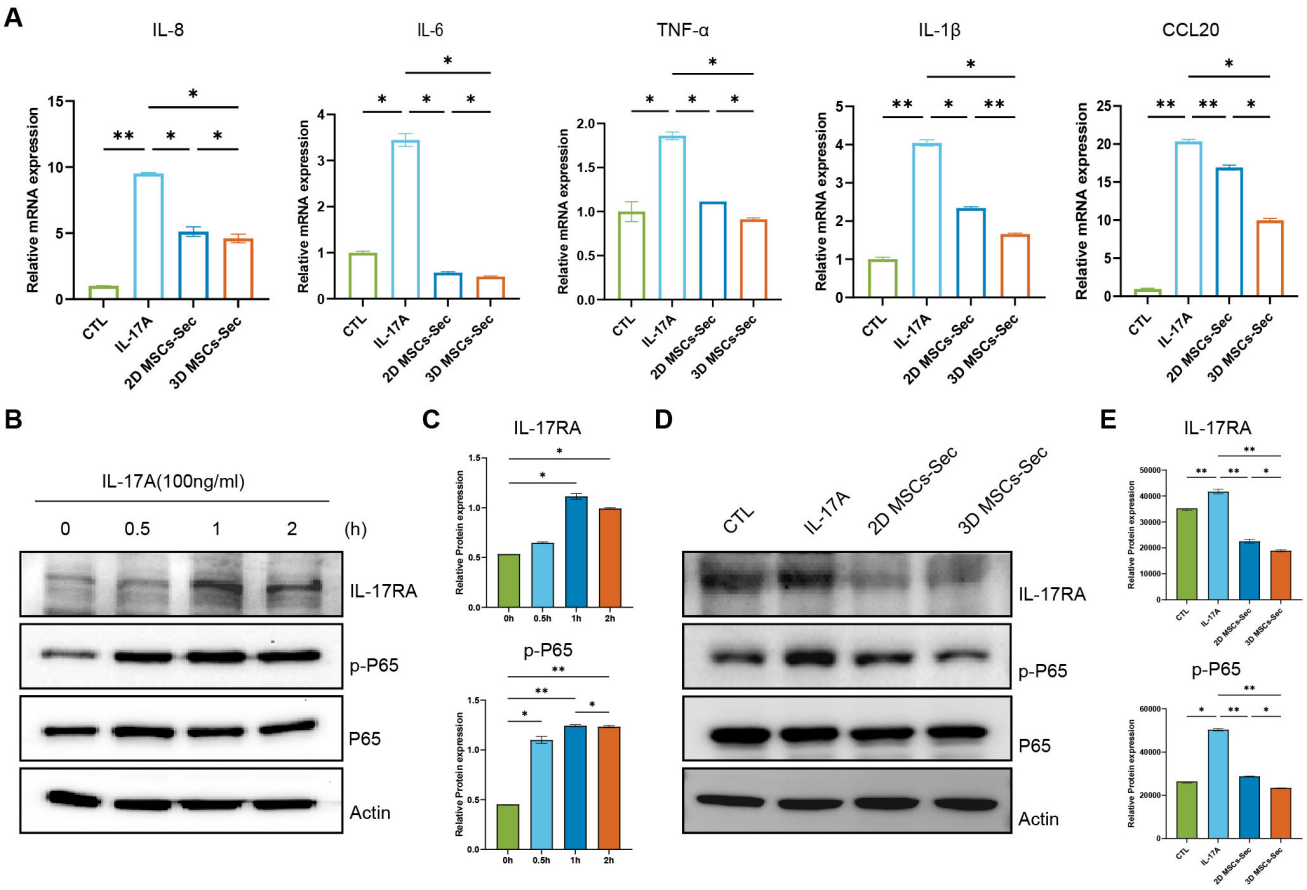
MSCs supernatants inhibited IL-17A-activated NF-κB signaling *in vitro*. **(A)** Hacat cells were pretreated with a fresh medium containing supernatants from hUC-MSCs in a 1:1 ratio, either in 2D or 3D culture, for 24 h before stimulation with IL-17A (100 ng/mL) for an additional 2 h. RT-qPCR analysis of mRNA levels of the IL-8, IL-6, TNF-α, IL-1β, and CCL20 in HaCaT cells. **(B, C)** Western blot analysis of the activation of IL-17RA, p-P65, and P65 in HaCaT cells stimulated with IL-17A at indicated time points. Actin was used as a loading control. Relative protein expression was normalized to that of the internal control. **(D, E)** Western blot analysis of IL-17RA, p-P65, and P65 in the four groups. Actin was used as a loading control. Relative protein expression was normalized to that of the internal control. Data are expressed as mean ± SEM. Statistical significance was calculated by one-way ANOVA with Tukey’s *post hoc* test. *P < 0.05, **P < 0.01, ns, not significant. *n* = 3 independent assays, with each experiment comprising 3 technical replicates for every condition.

MSCs improve therapeutic outcomes through modulating paracrine signaling and facilitating immunomodulation (20). IL-17A is known to stimulate the production of immune-related molecules such as cytokines and chemokines, which in turn induce inflammation in keratinocytes within psoriatic lesions. An *in vitro* psoriasis keratinocyte model was established by exposing keratinocytes to IL-17A to simulate certain aspects of psoriasis. To investigate whether hUC-MSCs supernatant reduces cytokine and chemokine production in keratinocytes after IL-17A stimulation, we analyzed cytokine and chemokine mRNA expression using RT-qPCR. The supernatant from 3D-hUC-MSCs significantly decreased the mRNA expression of IL-8, IL-6, IL-1β, TNF-α, and CCL20, which are upregulated by IL-17A in keratinocytes, compared with the supernatant from 2D-hUC-MSCs (**Figure 5A**). IL-17A enhanced the IL-17RA and NF-κB pathways in keratinocytes (**Figures 5B, C**).

To determine the activation of the NF-κB signaling pathway and understand the mechanism by which MSCs supernatants mitigate inflammation, we performed western blot analysis. Keratinocytes were stimulated with IL-17A in the presence or absence of MSCs supernatant, and the activation of p-P65 was assessed. The protein levels of IL-17RA and p-P65 were found to be elevated as a result of exposure to IL-17A. However, treatment with the supernatant from 3D-hUC-MSCs significantly reduced their levels in keratinocytes (**Figures 5D, E**). The findings suggest that the supernatant from 3D-hUC-MSCs more effectively reduces the inflammatory response in keratinocytes compared with the supernatant from 2D-hUC-MSCs by downregulating cytokine and chemokine production and inhibiting the IL-17RA and NF-κB pathways.

### 3.5 3D-hUC-MSCs significantly reduced psoriasis-like inflammation *in vivo*

**Figure 6:**
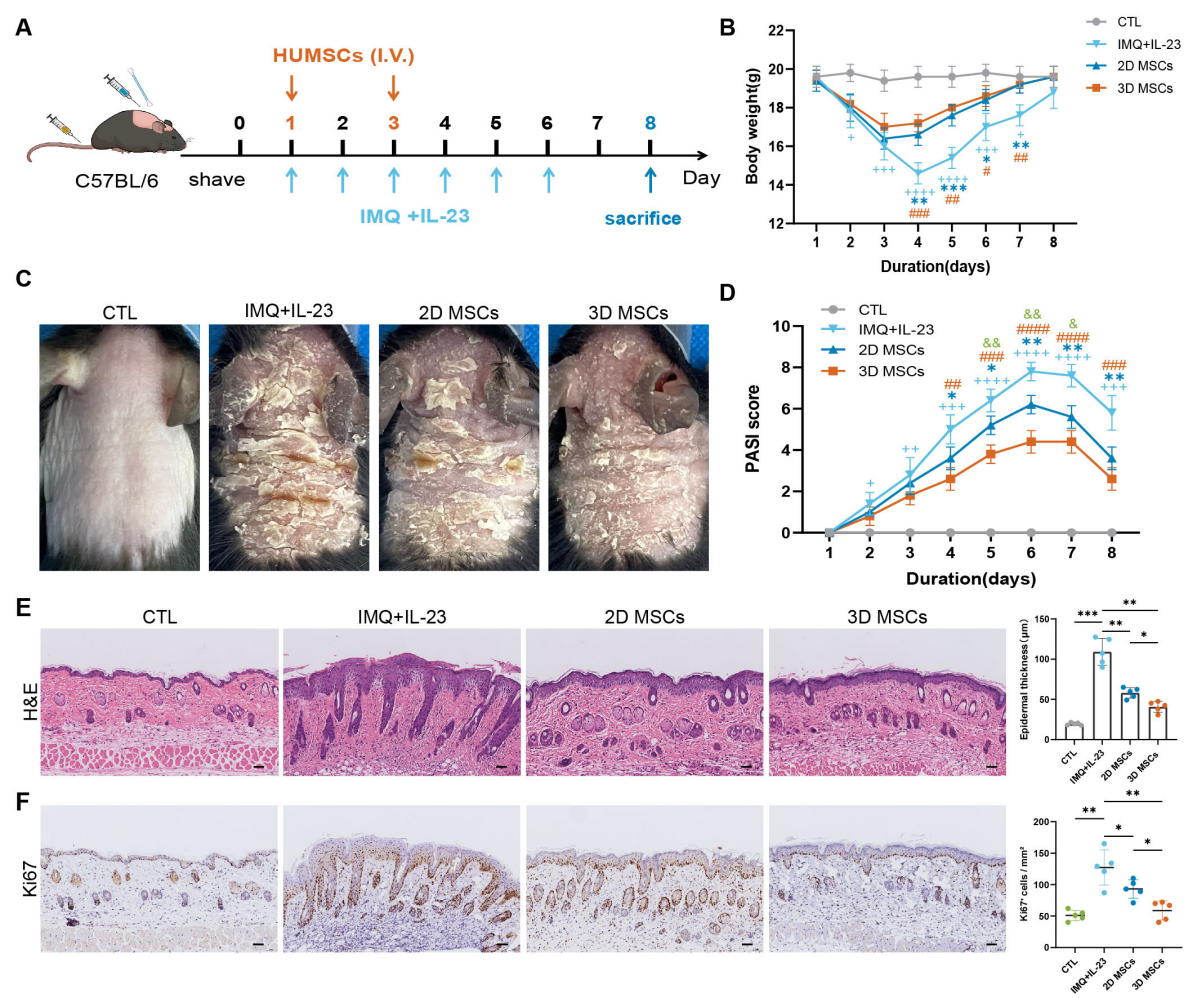
3D-hUC-MSCs improved morphological and histological characteristics of mice with IMQ + IL-23-induced psoriatic dermatitis. **(A)** The animal experiment protocol for the Control, IMQ + IL-23, IMQ + IL-23 + 2D-hUC-MSCs, and IMQ + IL-23 + 3D-hUC-MSCs groups (*n* = 5 per group). **(B)** The body weights of the four groups of mice. **(C)** Representative macroscopic view of dorsal skin in C57BL/6 mice on the 7th day after treatment. **(D)** Epidermal erythema, scaling, and thickening of the skin on the back were assessed daily. PASI scores were calculated by summing the scores of three independent criteria obtained for erythema, skin scaling and thickening (ranging from 0 to 12). **(E)** Representative images of H&E staining; scale bar: 50 µm. Quantitative analysis of epidermal thickness is shown in the accompanying bar graph. **(F)** Representative immunohistochemical images of Ki67 in dorsal skin samples from four groups of mice; scale bar: 50 µm. Quantification of Ki67-positive cells is shown in the bar graph. Data are represented as the mean ± SEM. Statistical significance was calculated by one-way ANOVA with Tukey’s *post hoc* test. +, ++, +++, ++++ vs. control group; *, **, *** vs. 2D MSCs group; #, ##, ###, #### vs. 3D MSCs group; &, && for 2D MSCs vs. 3D MSCs. P < 0.05, 0.01, 0.001, 0.0001; ns, not significant.

Following the implementation of routine quality assessment and *in vitro* experiments, there is a pressing need for the development of broader characterization metrics to accurately predict hUC-MSCs-based treatment outcomes. Toward this aim, experiments were conducted in a psoriasis mouse model according to the design shown in (**Figure 6A**). Mice were shaved on their backs and consecutively treated with IMQ and subcutaneous IL-23 injections for 6 days. Treatments included intravenous injections of saline, 2D-hUC-MSCs, or 3D-hUC-MSCs on days 1 and 3 (daily dose = 5 × 10^5^ cells per mouse). Compared with the control group, mice treated with 3D-hUC-MSCs showed a significant improvement in weight loss compared with mice treated with saline or 2D-MSCs (**Figure 6B**). In addition, IMQ + IL-23 treatment resulted in pronounced erythema and scaling of the skin, whereas phenotypic changes were significantly improved in the 2D-hUC-MSCs and 3D-hUC-MSCs groups (**Figure 6C**). 3D-hUC-MSCs had a more significant therapeutic impact than 2D-hUC-MSCs. Clinical changes in the four groups were evaluated daily using average Psoriasis Area and Severity Index (PASI) scores. The IMQ + IL-23 treatment led to evident erythema and scaling of the skin compared with the control group; administration of 3D-hUC-MSCs significantly alleviated these symptoms (**Figure 6D**). H&E staining revealed significant thickening of the epidermal layer and infiltration of inflammatory cells in the dermal layer of the IMQ+IL-23 group. By contrast, the 3D-hUC-MSCs groups showed improvements in skin pathology compared with the IMQ+IL-23 group, which was confirmed by quantitative analysis (**Figure 6E**). In immunohistochemical (IHC) analysis of Ki67 expression, a marker for proliferating cells, Ki67 expression was significantly reduced after 3D-hUC-MSCs treatment compared with the IMQ+IL-23 group, as supported by quantitative analysis of the staining (**Figure 6F**). These results indicate that 3D-hUC-MSCs were more effective in alleviating IMQ + IL-23-induced psoriasis-like lesions.

### 3.6 The identification of IL-17/NF-kB as a key target for the regulation of 3D-hUC-MSCs using RNA-seq

**Figure 7:**
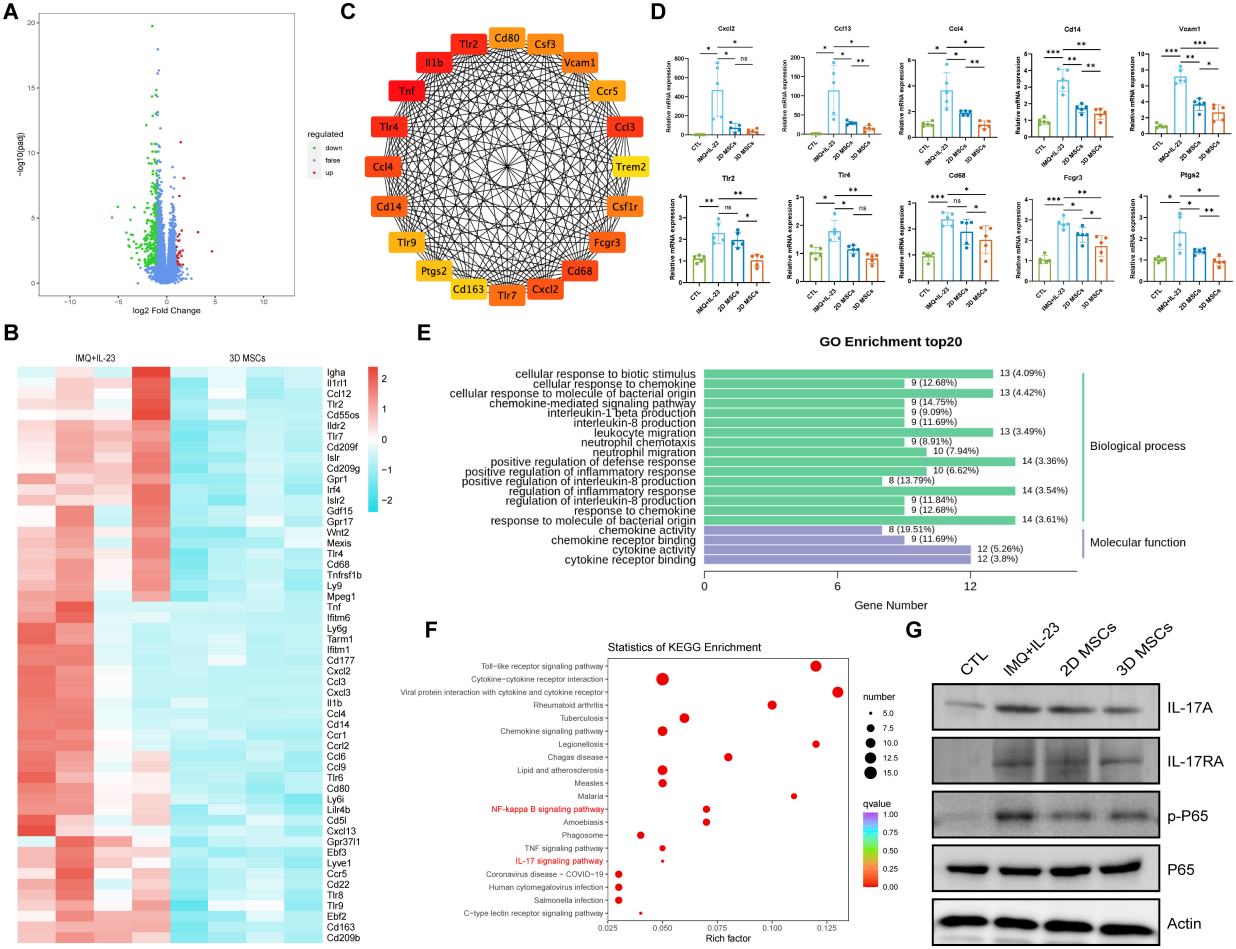
3D-hUC-MSCs treatment reverses psoriasis-like skin lesions by inhibiting IL-17/NF-kB signaling. **(A)** Volcano plots of downregulated (green) and upregulated (red) DEGs between 3D-hUC-MSCs-treated/untreated psoriasis-like skin lesions. **(B)** Heatmap of downregulated DEGs associated with inflammatory responses. **(C)** The 20 hub genes of the significantly downregulated DEGs. **(D)** The expression level of the top 10 genes verified by RT-qPCR. **(E)** DEGs classified based on their gene ontology (GO) terms for biological process (BP) and molecular function (MF). **(F)** KEGG pathway analysis of DEGs (FDR < 0.1). **(G)** Western blot analysis of IL-17A, IL-17RA, P65, and p-P65 in the four groups. Data are represented as mean ± SEM (*n* = 3). Statistical significance was calculated by one-way ANOVA with Tukey’s *post hoc* test. *P < 0.05, **P < 0.01, ***P < 0.001, ns, nonsignificant.

To further elucidate the molecular mechanisms underlying the disruption of IMQ + IL-23-induced psoriasis development by MSCs, we used RNA sequencing (RNA-seq) to compare gene alterations between the IMQ + IL-23-treated and 3D-hUC-MSCs-treated groups. A volcano plot showing the changes in differentially expressed genes (DEGs) after 3D-hUC-MSCs treatment is shown in (**Figure 7A**). Compared with the IMQ + IL-23-treated group, 324 genes were significantly altered in the 3D-hUC-MSCs group; 291 were down-regulated and 33 were up-regulated (fold change ≥ 2 and P < 0.05). Downregulated genes were closely associated with inflammation (**Figure 7B**). Protein-protein interaction (PPI) network analysis identified potential interactions among the down-regulated DEGs, suggesting that these genes may co-regulate inflammatory responses and immune regulation through interactions involved in the pathological process of psoriasis (**Figure 7C**). RT-qPCR verified the down-regulated expression of the top ten key genes, further supporting their importance in 3D-hUC-MSCs treatment (**Figure 7D**). In light of the biological significance of these findings, 3D-hUC-MSCs treatment may suppress inflammatory responses by down-regulating key inflammatory and immune-related genes, thereby alleviating psoriasis symptoms.

GO enrichment analysis showed that the down-regulated DEGs were mainly involved in biological processes related to signal transduction and the innate immune response (**Figure 7E**). KEGG pathway analysis revealed that the DEGs participated in several signaling pathways, such as Toll-like receptor signaling, cytokine-cytokine receptor interaction, NF-κB signaling, TNF signaling, and IL-17 signaling (**Figure 7F**). The protein expression levels of IL-17A, IL-17RA, and p-P65 demonstrated significant reductions, confirming that 3D-hUC-MSCs could ameliorate IMQ + IL-23-induced psoriasis-like skin inflammation in mice by affecting IL-17/NF-κB signaling (**Figure 7G**). These results indicate that IL-17-associated inflammatory genes and IL-17/NF-κB signaling are regulatory targets of 3D-hUC-MSCs in IMQ+IL-23-induced psoriasis-like inflammatory skin.

### 3.7 3D-hUC-MSCs improved morphological and histological features in mice with IMQ + IL-23-induced psoriasis-like dermatitis

**Figure 8:**
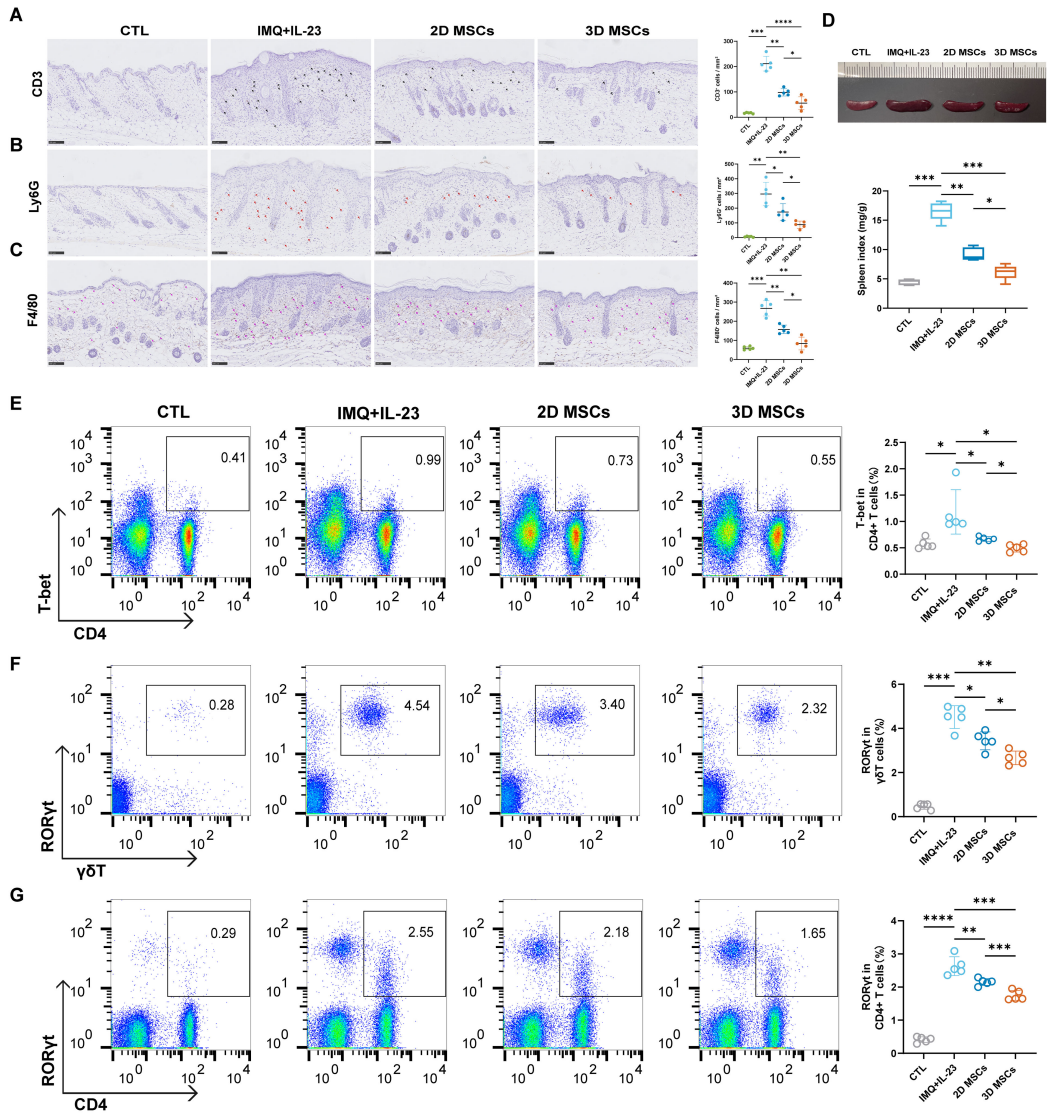
3D-hUC-MSCs reshaped the inflammatory ecosystem in the skin of psoriasis-like dermatitis model mice. **(A–C)** Infiltration of CD3, Ly6G, and F4/80 in skin tissue sections of the four groups measured by immunohistochemical staining; scale bar: 50 µm. Quantitative analysis of cell infiltration is shown in the accompanying bar graphs. **(D)** Spleens from the four groups of mice were isolated and weighed, and the spleen weight index was calculated as the organ weight per mouse body weight. **(E–G)** Representative quantitative results of T-bet+/CD4+, RORγt+/γδT+, and RORγt+/CD4+ cells in lymph nodes of different treatment groups analyzed by flow cytometry (n = 5). Data are represented as the mean ± SEM (*n* = 5). Statistical significance was calculated by one-way ANOVA with Tukey’s *post hoc* test. *P < 0.05, **P < 0.01, ***P < 0.001, ****P < 0.0001, ns, not significant.

**Figure 9:**
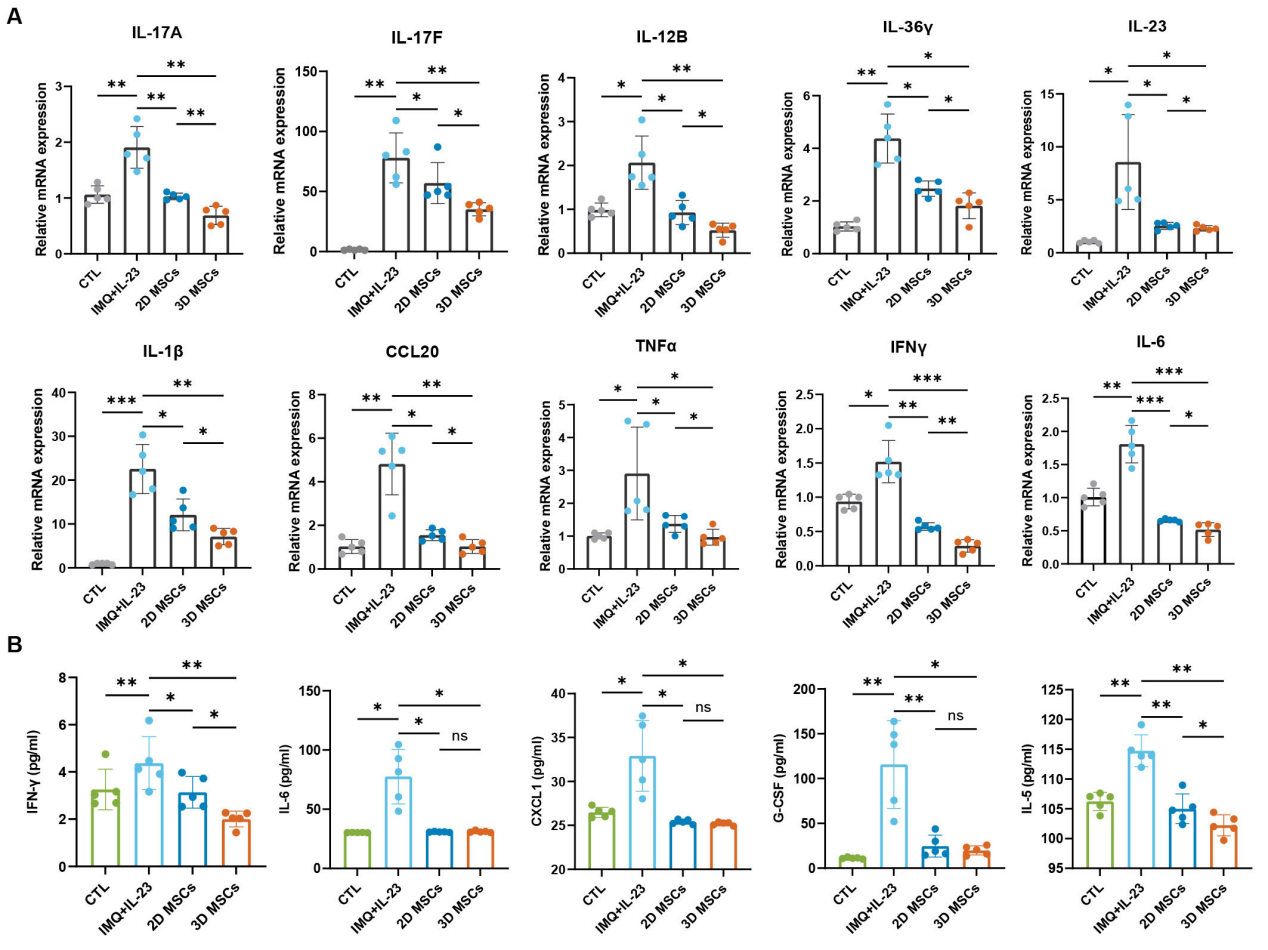
3D-hUC-MSCs reduced pro-inflammatory cytokine expression levels in psoriasis-like dermatitis model mice. **(A)** Relative mRNA levels of Il-17A, Il-17F, IL-12B, IL-23, IL-36γ, IL-6, Il-1β, TNF-α, IFN-γ, and CCL20 detected in skin tissues from various treatment groups using RT-qPCR (n =5). **(B)** ELISA of the serum expression levels of IFN-γ, IL-6, CXCL1, G-CSF, and IL-5 in different treatment groups (*n* = 5). Data are represented as mean ± SEM. Statistical significance was calculated by one-way ANOVA with Tukey’s *post hoc* test. *P < 0.05, **P < 0.01, ***P < 0.001, ns, not significant.

To determine the mechanisms by which 3D-hUC-MSCs provide relief for psoriasis, we conducted an assessment of the skin ecosystem in mice on day 8. Immunohistochemical analysis showed that 3D-hUC-MSCs significantly reduced CD3, Ly6G, and F4/80 cell infiltration in mouse skin treated with IMQ + IL-23, which was further supported by quantitative analysis (**Figures 8A–C**). The spleen index, which measures the ratio of spleen weight to body weight and serves as an indicator of inflammation severity, decreased in mice treated with 3D-hUC-MSCs (**Figure 8D**). The frequency and activation of different immune components were also examined through flow cytometry analysis, and T-bet and RORγT producing T cells were detected in mice after various treatments. There was a considerable decrease in the percentage and absolute number of T-bet+/CD4+, RORγT+/CD4+, and RORγT+/γδT+ cells in 3D-hUC-MSCs-treated mice compared with control mice (**Figures 8E–G**). These results suggest that 3D-hUC-MSCs might inhibit the proliferation of T cells and regulate Th1 and Th17 cell responses in the skin of IMQ + IL-23-induced psoriasis mice. Measurement of mRNA levels in skin tissues, including Il-17A, Il-17f, Il-12B, Il-23, Il-36γ, Ifn-γ, Il-6, Tnf-α, Il-1β, and CCL20, indicated that 3D-hUC-MSCs treatment decreased the production of pro-inflammatory cytokines and chemokines (**Figure 9A**); this reduction is consistent with the levels of cytokines in skin and serum (**Figure 9B**). These findings suggest that 3D-hUC-MSCs effectively reduced infiltration of immune cells and stimulated immunomodulatory cells in the skin and peripheral circulation, resulting in significant suppression of the inflammatory response.

## 4 Discussion

MSCs, which are highly valued for their pluripotency, have garnered significant attention in the field of stem cell research in recent years. Although the 2D culture method is widely used for MSC culture, it has several limitations, including restricted proliferative capacity and early senescence, as the pluripotency and differentiation potential of MSCs decrease with each successive culture passage. In addition, the conventional 2D culture system fails to replicate the natural microenvironment of living tissues, including their structural, physiological, and biochemical signals, as well as cell-substrate interactions, restricting the in-depth study and further development of MSCs (21, 22). To address these limitations, researchers are currently developing novel *in vitro* 3D cell culture systems (23). Recent studies have shown that the pluripotency and differentiation potential of MSCs are significantly enhanced in 3D culture (24). This culture system provides an ecological niche that closely mimics the *in vivo* environment, regulating many biological properties through direct cell-to-cell interactions. 3D culture can be performed in either scaffoldless (i.e., sphere) or scaffold-based structures (i.e., a hydrogel as a support for a hard polymer material) (25). However, due to limited oxygen and nutrient supply in 3D culture, cells may form aggregates and develop necrotic cores after prolonged incubation (26). To mitigate these issues, this study employed a rigid polymer scaffold-based 3D culture system to investigate the factors influencing the basic properties, stemness, and differentiation potential of MSCs. This system utilizes a rigid polymer scaffold, which is ablated before being added to a monolayer culture. This approach provides an opportunity for a deeper understanding of the impact of the new culture system on MSCs behavior and properties.

The International Society for Cell and Gene Therapy (ISCT) requires MSCs to be deficient in CD45 as well as express CD73, CD90, and CD105; these markers are crucial in cell and gene therapy research as they help to ensure purity and confirm the identity of MSCs (27). In our study, we found that hUC-MSCs were negative for the expression of CD45, CD14, CD19, CD34, and HLA-DR markers, and positive for the expression of CD73, CD90, and CD105 in both 2D and 3D cultures, showing that they meet these ISCT guidelines.

We compared hUC-MSCs in 2D and microcarrier-based 3D culture conditions and observed several significant differences in terms of cell morphology, gene expression, cell stemness, differentiation potential, and immunomodulation. The cells cultured in 3D conditions also maintained a stable expansion ploidy and exhibited a younger cell morphology. 3D-hUC-MSCs were lysed and digested, and then seeded into single-layer culture flasks, where they exhibited strong adhesion ability. Cells were spindle-shaped and smaller in size; several other morphologies have been obtained by different research groups. A recent study observed small, round, and spindle-shaped cells after 3D-cultured cells were re-monolayered (28). In addition, flat and large cell morphologies are considered to be associated with a more rapid senescence process and loss of stemness (29). Recent studies have shown diverse results regarding cellular senescence achieved in 3D culture by different research groups. Previous study found that the number of senescent cells in 3D-cultured Wharton’s jelly MSCs (WJ-MSCs) was lower than that in the 2D culture group (30). Conversely, a significant number of senescent cells were observed in long-term 3D cultures of AD-MSCs spheroids, although they were still fewer compared with those in 2D monolayer cultures (31). Some studies have reported higher rates of cell senescence under three-dimensional culture conditions (32). This variation may be attributed to factors such as the specific experimental conditions, culture system, oxygen concentration, nutrient supply, and cell types used. In our study, we measured β-galactosidase activity and found a significant reduction in the number of senescent cells in the 3D culture group compared with the 2D culture group. In addition, key senescence markers, including PARP1, P19, P21, and P53, were significantly down-regulated in the 3D culture group. This indicates that 3D culture offers a more intricate environment for cell growth, positively impacting stem cell morphology and reducing senescence, highlighting the significance of developing 3D culture systems for maintaining healthy cell growth.

Chromosomal fragile sites (CFSs) are regions on chromosomes that are sensitive to environmental stresses and cell physiology; these sites can cause chromosomal rearrangements, such as insertions, deletions, or inversions (33). Studies have shown that hypoxic environments can increase the expression of CFSs, resulting in chromosomal abnormalities (34). Research has shown that culturing hMSCs under hypoxic conditions can result in frequent breakage of chromosomes at CFSs (35). Our study found that the chromosomal karyotypes of the cells remained stable under both 2D and 3D culture conditions with no observable chromosomal abnormalities, implying that the 3D culture method is effective in preserving the genetic stability of stem cells, which is beneficial for various research and therapeutic applications.

Different culture conditions can lead to variations in growth factors, activation of signaling pathways, and cell interactions, resulting in differences in pluripotency and differentiation potentials. SOX2, OCT4, and NANOG are important markers related to stem cells and embryonic development. Recent studies have demonstrated that mesenchymal stem cells (MSCs) grown in 3D cultures exhibit significantly enhanced expression of pluripotency genes and differentiation compared with those grown in 2D cultures (36). For instance, the expression of OCT4, SOX2, and NANOG was significantly increased in cells cultured in 3D on fibrin scaffolds (37). In this study, we discovered that the mRNA expression of pluripotency genes was significantly increased under microcarrier-based 3D culture conditions. The regulatory network of these genes collectively maintains the pluripotent state of MSCs, enabling them to differentiate into various cell types, such as osteoblasts, adipocytes, and chondrocytes. In addition, hASCs cultured under 3D conditions exhibited enhanced pluripotency expression and an increased ability to differentiate into osteoblasts and chondrocytes (38). Previous studies have shown that cells cultured under 3D conditions have a greater potential for adipogenic and osteogenic differentiation compared with those cultured under 2D conditions (39). Our study further observed that umbilical cord MSCs exhibited enhanced adipogenic, chondrogenic, and osteogenic differentiation under microcarrier-based 3D culture conditions, indicating an increase in their pluripotency and differentiation potential.

The enhanced therapeutic effects observed in 3D-hUC-MSCs may be attributed to several biological changes induced by the microcarrier-based 3D environment. Accumulating evidence suggests that 3D culture reshapes the MSC secretome, promoting increased secretion of anti-inflammatory cytokines, such as IL-10 and TGF-β, as well as growth factors, and extracellular vesicles enriched with immunoregulatory cargos (40, 41). In addition, the 3D microenvironment supports stronger cell-cell and cell-matrix interactions, which in turn activate stemness-associated transcriptional programs, reduce oxidative stress, and improve cellular metabolic fitness (42, 43). Together, these alterations enhance the immunomodulatory functions of MSCs, including their ability to suppress Th17-driven inflammation, attenuate TNF-α-mediated responses, and inhibit neutrophil activation. Therefore, the superior therapeutic outcomes observed in our psoriasis model may be closely linked to these 3D-induced changes in the secretory profile and functional properties of MSCs.

MSCs therapy has been proposed as a novel cell therapy for treating inflammatory and immune-mediated diseases (44). Psoriasis is an immune-mediated chronic inflammatory skin disease, although its precise pathogenic mechanisms remain incompletely understood, numerous preclinical studies and clinical trial results have demonstrated the potential of stem cells in psoriasis treatment. By comparing our study with previous research outcomes, it becomes evident that 3D cultured MSCs exhibit unique advantages in treating psoriasis. The efficacy of conventional 2D-cultured MSCs has been affirmed in animal models and human patients. Previous studies have found significant therapeutic effects of human embryonic stem cell-derived MSCs (hE-MSCs) in an IMQ-induced psoriasis mouse model, as evidenced by decreased clinical skin scores and reduced epidermal thickness (45). Another study also found that psoriasis was successfully ameliorated in a mouse model by administration of human umbilical cord-derived mesenchymal stem cells (hUC-MSCs), possibly by inhibiting neutrophil function and type I interferon production by plasmacytoid dendritic cells (46). These studies indicate a certain degree of efficacy of 2D cultured MSCs in psoriasis treatment. However, our research utilizing 3D cultured hUC-MSCs demonstrated significant enhancements in cellular potency and anti-aging properties. Compared with 2D-hUC-MSCs, 3D-hUC-MSCs more effectively suppressed the infiltration of T cells, neutrophils, and macrophages in a psoriasis mouse model, attenuating psoriasis progression; in addition, 3D-hUC-MSCs exhibited more inhibition of differentiation of CD4+ T cells into Th1 and Th17 cells and significantly suppressed the inflammatory response of keratinocytes by modulating paracrine signaling, alleviating the expression of the IL-17/NF-kB signaling pathway. These findings demonstrate the advantages of 3D-hUC-MSCs in cellular functionality and therapeutic efficacy, as well as reveal their immense potential in practical applications for treating psoriasis and possibly other immune-mediated diseases.

Recently, local application of MSCs exosome preparations has been successfully conducted in mouse models. These exosomes possess immunomodulatory properties, reducing inflammation by enhancing the secretion of anti-inflammatory cytokines, promoting Treg polarization, and inhibiting complement activation. Local application of MSCs exosomes significantly reduced IL-17 and C5b-9 terminal complement activation complexes in the skin of mice in an IMQ-induced psoriasis mouse model (47). In our study, we also observed this phenomenon; compared with 2D culture, the conditioned medium of 3D cultured hUC-MSCs significantly inhibited the response of keratinocytes. However, further exploration is needed to determine which specific components play important roles.

The prospects of MSCs therapy for psoriasis and other skin diseases are promising. The application of 3D culture technology is expected to enhance the therapeutic efficacy of MSCs and reduce side effects. This technology can further optimize the stemness and immunomodulatory functions of MSCs, extending their application potential to other immune-mediated skin diseases such as atopic dermatitis and systemic lupus erythematosus. Through gene editing and molecular biology techniques, specific functions of MSCs, such as their anti-inflammatory properties and tissue repair capabilities, could be selectively enhanced, expanding their therapeutic potential. The results of this study support the potential of MSCs in psoriasis treatment, as well as emphasize the significant advantages of 3D-hUC-MSCs in therapeutic efficacy. These findings provide a solid scientific foundation for the future application of MSCs in dermatology, indicating that further exploration and development in this field will lead to more breakthroughs.

Our study demonstrates that microcarrier-based 3D culture enhances the proliferation, maintenance of stemness, and immunomodulatory functions of hUC-MSCs. This finding aligns with previous reports that highlight the beneficial effects of 3D culture on the biological performance of MSCs. In designing this study, we acknowledged the limitation of utilizing a single donor; thus, future investigations will incorporate samples from multiple donors to systematically evaluate how donor variability influences the functionality of 3D-cultured MSCs. We have extensively optimized and validated the 3D microcarrier system used in this study to support MSC expansion and functional maintenance. However, as the system progresses toward clinical-grade large-scale production, batch-to-batch consistency and process scalability remain a challenge which will be addressed in future scale-up studies. The IMQ + IL-23 model effectively replicates key immune pathological processes driven by the IL-23/IL-17 axis, primarily reflecting acute inflammation; however, it does not fully capture the chronic and recurrent characteristics of human plaque psoriasis. Future studies incorporating additional psoriasis models will be essential for further evaluating long-term efficacy and the underlying mechanisms involved.

## 5 Conclusion

**Figure 10:**
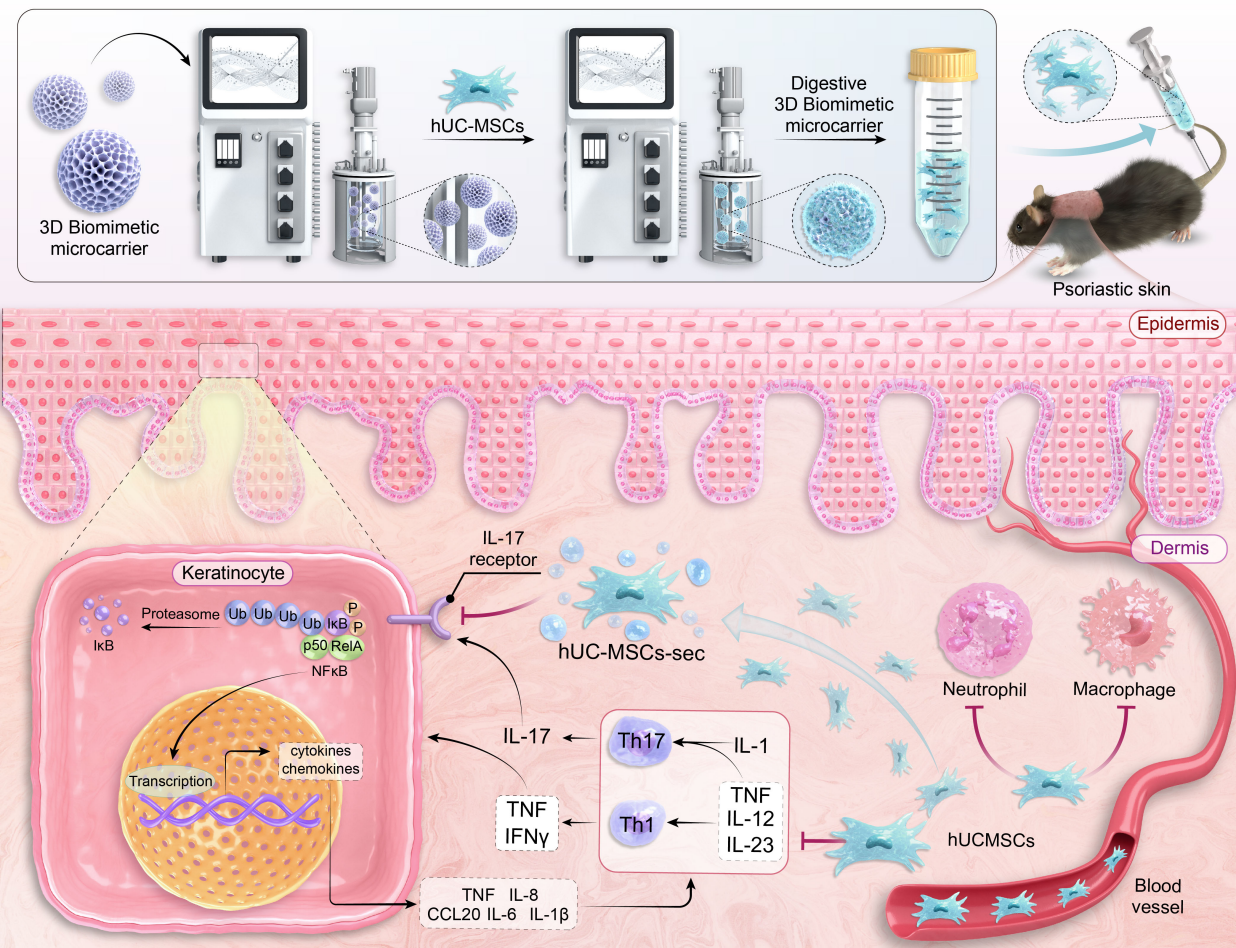
Schematic of 3D biomimetic microcarrier-expanded human umbilical cord-derived mesenchymal stem cells (hUC-MSCs) for the treatment of psoriasis and their potential mechanisms.The upper panel illustrates the expansion and collection of hUC-MSCs using a 3D biomimetic microcarrier–bioreactor system and their subsequent application in a psoriasis model. The lower panel depicts the potential mechanisms by which hUC-MSCs and their secretome (hUC-MSCs-sec) act within the psoriatic inflammatory microenvironment. hUC-MSCs primarily suppress the activation of Th1 and Th17 cells, while also reducing inflammation and tissue infiltration by neutrophils and macrophages, leading to a decrease in pro-inflammatory cytokines. The secretome of hUC-MSCs attenuates keratinocyte activation by inhibiting the IL-17A/NF-κB signaling pathway. In turn, inflammatory mediators and chemokines produced by keratinocytes can stimulate Th1/Th17 cells and neutrophils, creating a feedback loop. The combined, multi-targeted actions of hUC-MSCs and their secretome disrupt this cycle, thereby alleviating psoriatic skin inflammation.

This study demonstrated that microcarrier-based 3D culture conditions significantly enhanced the stem cell properties of hUC-MSCs, including pluripotency, immunomodulatory factors, and mesodermal differentiation potential. In addition, adipose, cartilage, and bone differentiation potentials of MSCs were enhanced under 3D culture conditions. Furthermore, this study revealed that the IL-17/NF-κB signaling pathway is involved in the effect of MSCs on psoriasis in mice, with 3D-hUC-MSCs being more effective in suppressing the inflammatory response of skin lesions than 2D-hUC-MSCs. The significance of 3D-hUC-MSCs lies in their ability to alleviate the inflammatory response involved in psoriatic lesions by reducing immune cell infiltration and modulating the expression of inflammatory factors and chemokines. The microcarrier-based 3D culture method is an effective strategy for producing high-quality MSCs with a wide range of potential applications; it is expected to play an important role in autoimmune disease treatment, regenerative medicine, drug discovery, and tissue engineering (**Figure 10**).

## Data Availability

The datasets presented in this study can be found in online repositories. The names of the repository/repositories and accession number(s) can be found below: https://www.ncbi.nlm.nih.gov/bioproject/, PRJNA1153642.

## Supplementary material

Supplementary Table 1 Primer sequences for real-time quantitative PCR.
