# Supplementary material for: Evaluation of 3D biomimetic microcarriers for enhancing therapeutic efficacy of human umbilical cord mesenchymal stem cells in psoriasis treatment
Source: Front Immunol. 2026 Feb 3;17:1687424. doi: 10.3389/fimmu.2026.1687424 (PMC12909563; doi:10.3389/fimmu.2026.1687424)
Supplement: Supplementary Table 1 — Primer sequences for real-time quantitative PCR. [file Table1.pdf]

Table S1. Primer sequences for real-time quantitative PCR.

	Forward 5'-3'	Reverse 5'-3'
Homo GAPDH	GCATCTTCTTTTGCGTCG	TGTAAACCATGTAGTTGAGGT
Homo SOX2	TGGCGAACCATCTCTGTGGT	CCAACGGTGTCAACCTGCAT
Homo OCT4	TCGAGAAGGATGTGGTCCGA	GCCTCAAAATCCTCTCGTTG
Homo Nanog	CCTGTGATTTGTGGGCCTG	GACAGTCTCCGTGTGAGGCAT
Homo P53	CAGCACATGACGGAGGTTGT	TCATCCAAATACTCCACACGC
Homo P21	CGATGGAACTTCGACTTTGTCA	GCACAAGGGTACAAGACAGTG
Homo P19	GATCCAGGTGGGTAGAAGGTC	CCCCTGCAAACTTCGTCCT
Homo PARP1	CGGAGTCTTCGGATAAGCTCT	TTTCCATCAAACATGGGCGAC
Homo IL-10	GACTTTAAGGGTTACCTGGGTTG	TCACATGCGCCTTGATGTCTG
Homo IL4	CCAACTGCTTCCCCCTCTG	TCTGTTACGGTCAACTCGGTG
Homo TGF-β	GGCCAGATCCTGTCCAAGC	GTGGGTTTCCACCATTAGCAC
Homo IL-8	GTGCAGTTTTGCCAAGGAGT	AAATTTGGGGTGGAAAGGTT
Homo IL-6	TGCAAGAGACTTCCATCCAGT	CTGCAAGTGCATCATCGTTGT
Homo IL-1β	GCCACCTTTTGACAGTGATG	AAGGTCCACGGGAAAGACAC
Homo CCL20	CAGGCAGAAGCAAGCAACTAC	AGCTTCATCGGCCATCTGTC
Homo TNF-α	TAGCCCACGTCGTAGCAAAC	TAGCAAATCGGCTGACGGTG
Mus IL-23	CACCAGCGGGACATATGAATCTAC	CTGTTGTCCTTGAGTCCTTGTGG
Mus TNF-α	GTGCCTATGTCTCAGCCTCTTCTC	GTTTGTGAGTGTGAGGGTCTGG
Mus IL-1β	GCATCCAGCTTCAAATCTCGC	TGTTCATCTCGGAGCCTGTAGTG
Mus IL-36γ	TGAGCCAATGAAGCCATTCCT	GGAAGATGGGGTTGCCAGTC
Mus IL-6	CCCCAATTTCCAATGCTCTCC	CGCACTAGGTTTGCCGAGTA
Mus CCL20	GTGGCAAGCGTCTGCTCTT	CAGTCGTAGTTGCTTGCTTCTG
Mus IFN-γ	CTCAAGTGGCATAGATGTGGAAG	TGACCTCAAACTTGGCAATACTC
Mus IL-17A	TCCACCGCAATGAAGACCCT	CATGTGGTGGTCCAGCTTTCC
Mus IL-17F	ATGAAGTGCACCCGTGAAACAG	CTGGAGCGGTTCTGGAATTCA
Mus IL-12b	ATTGAACTGGCGTTGGAAGC	GCGGGTCTGGTTTGATGATGT
Mus GAPDH	CCTCGTCCCGTAGACAAAATG	TGAGGTCAATGAAGGGGTCGT
