# Supplementary material for: Evaluation of 3D biomimetic microcarriers for enhancing therapeutic efficacy of human umbilical cord mesenchymal stem cells in psoriasis treatment
Source: Front Immunol. 2026 Feb 3;17:1687424. doi: 10.3389/fimmu.2026.1687424 (PMC12909563; doi:10.3389/fimmu.2026.1687424)

Supplementary Figure 1. Uncropped data for Figure 4B.

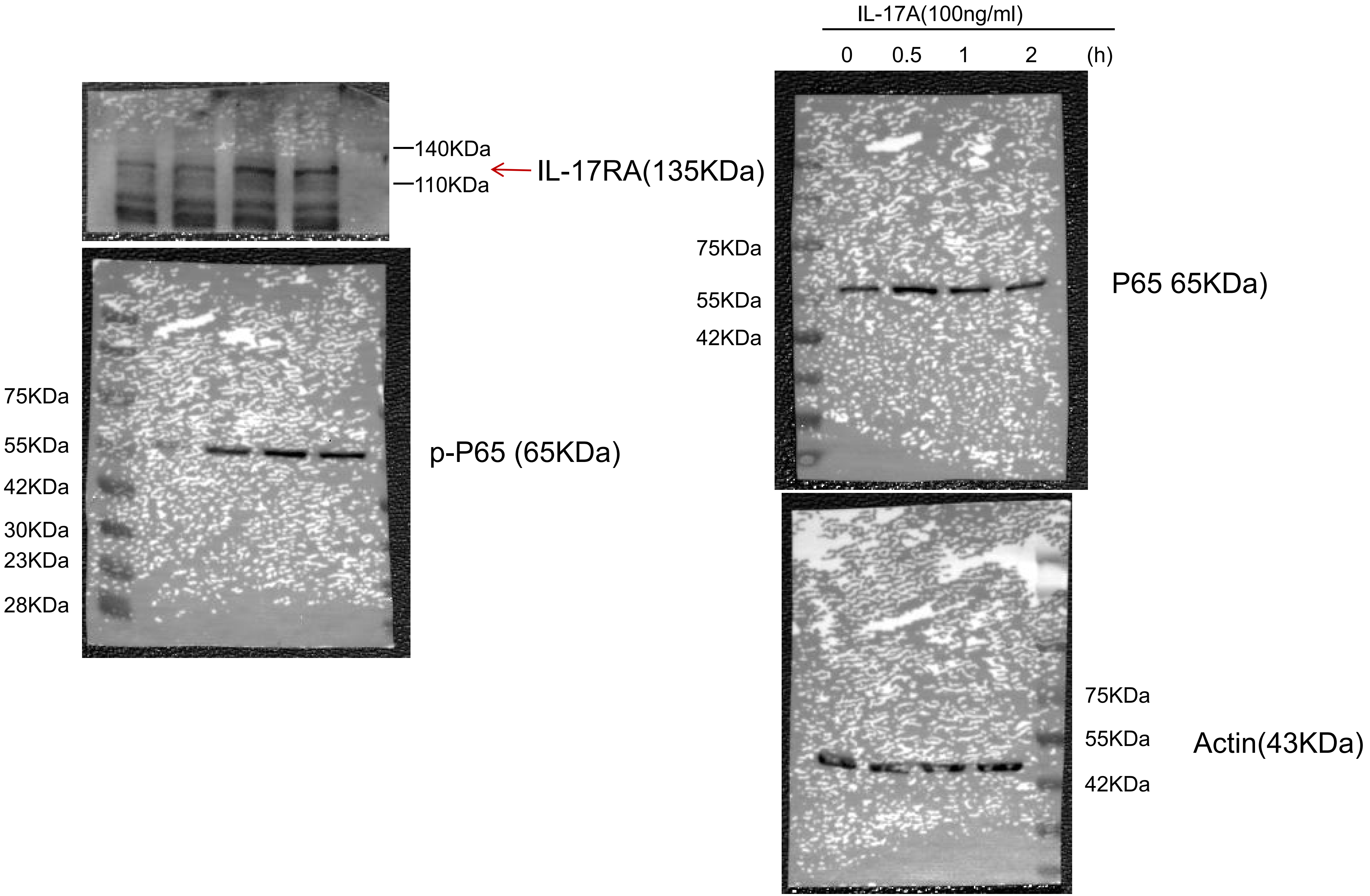

Supplementary Figure 2. Uncropped data for Figure 4D.

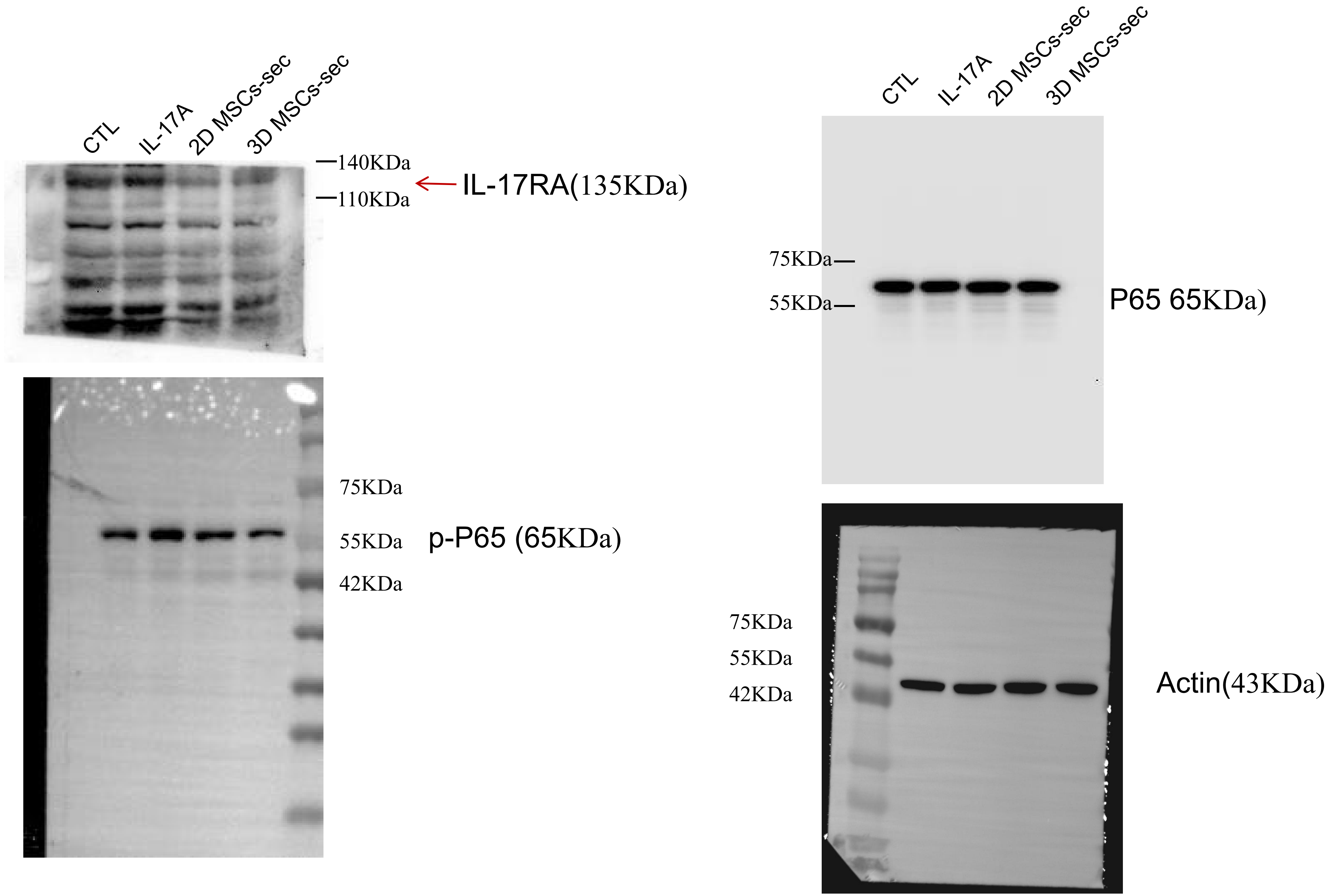

Supplementary Figure 3. Uncropped data for Figure 6G.

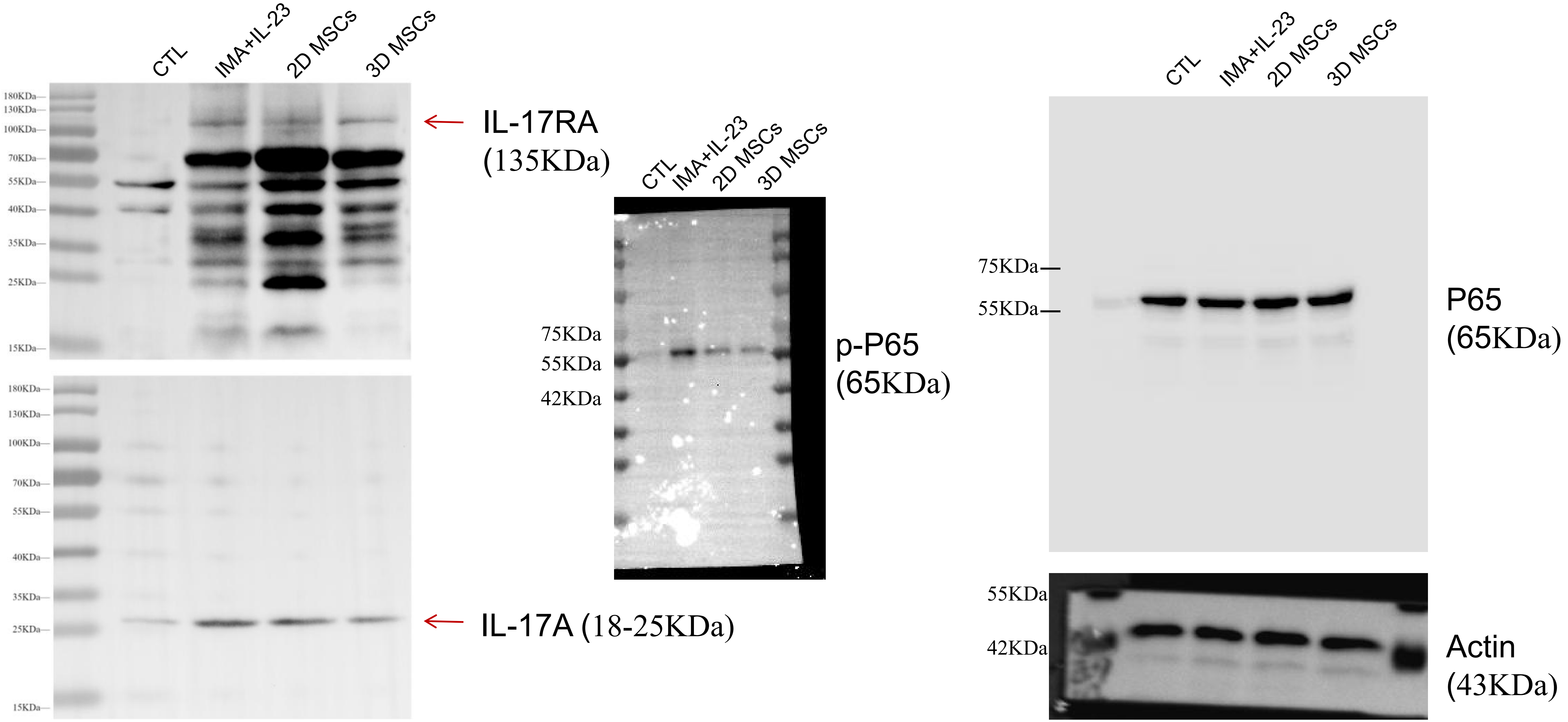

Supplement: Supplementary file 2 [file Supplementaryfile1.pdf]
